# Correction: Glutamate dehydrogenase (Gdh2)-dependent alkalization is dispensable for escape from macrophages and virulence of *Candida albicans*

**DOI:** 10.1371/journal.ppat.1009877

**Published:** 2021-08-30

**Authors:** Fitz Gerald S. Silao, Kicki Ryman, Tong Jiang, Meliza Ward, Nicolas Hansmann, Chris Molenaar, Ning-Ning Liu, Changbin Chen, Per O. Ljungdahl

After this article [1] was published, we discovered that the results reported for Gdh2-GFP in Figs 2–4 instead represented results from a strain expressing Put2-GFP. This error was discovered when validating a novel set of strains individually carrying GFP-tagged mitochondrial proteins. Strain CFG273 (*GDH2*/*GDH2-GFP*) as published, was used as a positive control. To our surprise and dismay, strain CFG219, constructed during the same period as CFG273, expressed a Put2-GFP protein that migrated precisely as the GFP-tagged construct expressed in CFG273. Subsequent Mass Spec analysis of immunoprecipitated GFP constructs (GFP-pulldowns) confirmed that the presumed Gdh2-GFP in CFG273 was in fact Put2-GFP. We traced this error to the freezing of strains, i.e., CFG219 was frozen twice, once as CFG273. The original CFG273 strain carrying a PCR-verified *GDH2-GFP* allele was thereby lost.

Since identifying this error, we reconstructed CFG273 using the purified *GDH2-GFP* tagging cassette that was originally used and fully verified the *GDH2-GFP* allele precisely as described (see the updated S1 Fig of this notice) and repeated the localization and expression experiments. Data confirming correct targeting and identification of the resultant strain are in S1 Fig of this notice. We provide here updated versions of Figs 2–4, S1 Vid and S2 Vid, that report results obtained using the correct Gdh2-GFP strain. For comparison, the updated figures and videos also retain the results, now correctly attributed to Put2-GFP. The results regarding Put2 have not previously been published and are of additive value to understanding proline metabolism in *C*. *albicans*.

The main results of the new, corrected experiments are:

- Based on localization of a Gdh2-GFP reporter protein, we provide evidence that Gdh2 is localized in the cytoplasm, instead of in the mitochondria as originally reported [1]. The updated results (Fig 2) indicate that Gdh2-GFP co-fractionates with the cytoplasmic protein Tdh3. Gdh2-GFP does not co-localize with MitoTrackerRed or mitochondrial localized Put1-RFP and Put2-GFP, and consistently, does not co-fractionate with Put1-RFP or Put2-HA.
- The expression of Gdh2-GFP is repressed by glucose (Fig 3C) and decreases as the pH of the media approaches neutrality (Fig 3B and 3D). In contrast the expression of Put2-GFP is not sensitive to pH and remains elevated. The apparent pH-dependent expression of Gdh2 suggests a regulatory mechanism that likely functions to limit further ammonia production and release, the consequence of Gdh2-catalyzed deamination of glutamate.
- Gdh2-GFP (cytoplasm) expression is induced similar to Put2-GFP (mitochondria) in cells engulfed by RAW264.7 macrophages (Fig 4).

Updated text is provided below for the Results subsections affected by the above errors. Note that in light of the updates to the figures, the title of the subsection, “Environmental alkalization originates in the mitochondria” is hereby updated to, “Environmental alkalization is linked to mitochondrial function”.

Although the main conclusions, regarding Gdh2 being the major source of ammonia and environmental alkalization, and that phagosomal alkalization is not required to induce-hyphal growth in macrophages, are unaffected by this correction, the correct assignment of Gdh2 as a cytoplasmic component is essential to fully understand its biological function. We apologize for the errors in the original publication and sincerely hope that research in other laboratories have not been adversely affected.

A member of *PLOS Pathogens*’ Editorial Board reviewed the updated figures and files and the article’s main conclusions are upheld.

## Updated text for affected Results subsections

### Environmental alkalization is linked to mitochondrial function (*formerly titled “Environmental alkalization originates in the mitochondria”*)

*The following is added as a second paragraph in this subsection*:

To examine how Gdh2-dependent alkalization is linked to mitochondrial function, we sought to determine where Gdh2 localizes. There is lack of consensus regarding Gdh2 localization in fungi, e.g., in *S*. *cerevisiae* Gdh2 has been reported to be a mitochondrial [2, 3] or cytosolic component [4–7]. Cells (CFG273) expressing the functional *GDH2-GFP* reporter were grown in synthetic glutamate medium with 0.2% glucose (SED_0.2%_) and YNB+CAA. Contrary to an earlier version of this publication, which mistakenly presented Put2-GFP as Gdh2-GFP, we now show that the *C*. *albicans* Gdh2 is a cytosolic enzyme (Fig 2B); Gdh2-GFP and MitoTracker (MTR) do not co-localize. This is in stark contrast to the overlapping pattern of Put2-GFP and MTR, confirming that Put2 is a mitochondrial enzyme [8]. We performed subcellular fractionation analysis using strains derived from the *GDH2-GFP* strain (CFG273) expressing either *PUT2-HA* or *PUT1-RFP* (Fig 2C). Put2 (Put2-HA) and Tdh3 (glyceraldehyde-3-phosphate dehydrogenase) served as *bona fide* mitochondrial and cytosolic markers, respectively. Consistent with the microscopy and the lack of a strong mitochondrial presequence at the N-terminus (Fig 2C) [9], Gdh2 co-localized with Tdh3 and not with Put2 or Put1.

### Gdh2 expression is repressed by glucose

To follow up on the observations that glucose negatively affects Gdh2 activity and that alkalization is dependent on mitochondrial function we sought to visualize cytosolic Gdh2 and mitochondrial-Put2 expression in living cells when shifted from repressing YPD (2% glucose) to non-repressing YNB+CAA. To do this, we used the same Gdh2-GFP and Put2-GFP reporter strains described earlier (Fig 2B). This enabled us to observe Gdh2 and Put2 expression at the single cell level over a period of 6 h in cells growing on a thin YNB+CAA agar slab. In both cases the GFP signal was initially weak (t = 0 h), becoming more intense as time progressed and as cells underwent several rounds of cell division (Fig 3A). These results strongly suggest that proline catabolism in the mitochondria generates the glutamate that serves as substrate of cytosol-localized Gdh2.

To relate this observation with the actual alkalization process, we analyzed the levels of Gdh2-GFP in cells grown in liquid culture taken at similar time points. Cells, pre-grown in YPD (2% glucose), were shifted to YNB+CAA and the levels of Gdh2-GFP were assessed by immunoblot analysis after 6 h of growth at 37 ^o^C (Fig 3B). The pH of cultures with a high starting cell density (OD_600_ = 2.0) increased and became more alkaline, whereas the pH of cultures with a low starting inoculum (OD_600_ = 0.05) remained acidic (Fig 3B, upper panels). Interestingly, the Gdh2 protein levels in cells under these two distinct conditions were significantly different; Gdh2 levels in cells obtained from the low starting inoculum and still acidic culture were notably higher (Fig 3B, lower panels).

Next, we examined the expression of Gdh2-GFP in cells shifted from YPD to YPG (Fig 3C; upper panels). The expression of Gdh2-GFP in cells grown in YPD was below the level of detection (Fig 3C, lanes 1, 7, 13–24). Four hours following the shift to YPG, Gdh2-GFP expression was detected (Fig 3C, lanes 3 and 9), reaching maximum levels at 6 h (Fig 3C, lanes 4–6, 10). The levels of Gdh2-GFP decreased 4 h after the addition of glucose (2% final concentration) (Fig 3C, lane 12). Similar to Gdh2, the basal expression of Put2 in YPD is low (Fig 3C, lower panel, lane 1), but is rapidly induced following the shift to glycerol, remaining high 4 h after the addition of glucose (2% final concentration) (Fig 3C, lower panels, lane 12). The latter observation is reminiscent of isocitrate lyase (Icl1), a glyoxylate cycle enzyme that is not subject to catabolite inactivation in *C*. *albicans* [10, 11]. These results are consistent with *GDH2* and *PUT2* expression being glucose-repressed.

To further investigate the effect of glucose on Gdh2-GFP expression, we shifted YPD grown cells to YNB+CAA (starting cell density of OD_600_ = 2) supplemented with 2% glucose, 0.2% glucose, 1% glycerol or no additional carbon source; the latter three conditions are not repressive to mitochondrial function [8]. As shown in Fig 3D (upper panels, lanes 1, 5, 9, and 13), the level of Gdh2-GFP in cells pre-grown in YPD is below the limit of detection. The expression of Gdh2-GFP in (YNB+CAA, no addition) peaked after 2 h and then gradually diminished. This pattern coincides with the appearance of degradation products (Fig 3D, asterisk) and the environmental pH becoming more neutral (Fig 3E), suggesting a regulatory axis between Gdh2 and pH. Consistent with pH affecting the stability of Gdh2, Gdh2 was found to be more highly expressed in cells growing in media under acidic compared to near neutral conditions (Fig 3B). Surprisingly, in contrast to YPD, we were able to detect basal expression of Gdh2 in the presence of 2% and 0.2% glucose, but the levels did not increase even after 6 h, suggesting that YPD contains additional component that contributes to repression of Gdh2 expression. We complemented this experiment with microscopy to visualize the expression of Gdh2 in wildtype cells (S6A and S6B Fig). Consistent with the results from the immunoblot analysis, we observed a significant increase in cytosolic fluorescence (measured as mean fluorescence intensity, MFI) (S6B Fig) after shifting the cells to YNB+CAA with or without 1% glycerol (S6A Fig) and then diminished after succeeding timepoints. The MFI of cells grown in YNB+CAA with 0.2% glucose, but not with 2% glucose, also increased. The expression kinetics differed from the expression of Put2-GFP. Unlike Gdh2 that is virtually repressed in YPD (Fig 3C), we observed a low basal expression of Put2-GFP in YPD-grown cells (t = 0 h) that dramatically increased during growth in YNB+CAA without or with alternative carbon source (1% glycerol) or reduced glucose (0.2%) (Fig 3D, lower panel). During the course of growth, the media became successively alkaline, increasing from the starting pH of 4 to 7 (Fig 3E). These results are consistent with significant increased mitochondria-localized Put2-GFP (S6A and S6B Fig). By contrast, cells grown in 2% glucose exhibited very weak fluorescence, but significantly higher than YPD-grown cells. The relatively slower appearance of Put2-GFP and Gdh2-GFP fluorescence in cells cultured in the presence of 0.2% glucose, compared to 1% glycerol or CAA alone, is attributed to a more gradual relief from the glucose repressing conditions of the YPD preculture. These results clearly demonstrate that both Gdh2 and Put2 expression are sensitive to repression by glucose.

Furthermore, these results suggest that glutamate, the product of Put2 catalysis in the mitochondria, is efficiently generated when glucose becomes limited. In addition, the finding that Gdh2 expression is induced in cells growing in media rich in amino acids (i.e., YNB+CAA or YPG) indicates that *GDH2* expression in *C*. *albicans*, in contrast to *S*. *cerevisiae* [12], is not subject to nitrogen catabolite repression (NCR). NCR is a supra-pathway that prevents the utilization of non-preferred nitrogen source when preferable nitrogen source such as amino acids or ammonium is available [13–15]. This occurs via nuclear exclusion of Gln3 and Gat1 GATA transcription factors from its target promoters.

### Gdh2-GFP expression is rapidly induced upon phagocytosis by macrophages

These results led us to evaluate the capacity of *gdh2*-/- cells to filament within phagosomes of engulfing macrophages. Here too, alkalization is believed to be essential for hyphal growth in the phagosome of engulfing macrophages. First, we determined whether Gdh2 expression is derepressed in phagocytized C. albicans cells. The Gdh2-GFP reporter strain (CFG273) was co-cultured with RAW264.7 (RAW) macrophages and phagocytosis events were followed by time-lapse microscopy. The Gdh2-GFP signal intensity increased upon phagocytosis, which remained stable as hyphae start to emanate from the mother cells (Fig 4B; SV1A Vid). Consistent to their activities being linked, the level of Put2-GFP (previously mistakenly reported as Gdh2-GFP) also increased following engulfment by macrophage (SV1B Vid). However, unlike the more stable Gdh2-GFP signal intensity, the Put2-GFP signal increased upon phagocytosis, peaking at around 1 h, and thereafter decreasing during hyphal extension. We repeated these experiments using primary murine bone marrow-derived macrophage (BMDM) and observed similar patterns of GFP fluorescence (SV2A and SV2B Vid), however, due to the inherent green autofluorescence of BMDM, the GFP fluorescence appeared less pronounced. These time-lapse images provide qualitative, yet strong evidence that Gdh2 and Put2 expression is induced upon macrophage engulfment; the Put2 result is consistent to our previous data obtained by immunocytochemistry using Put2-HA construct [8]. Attempts to quantify the observed fluorescent signals in the limited number of phagocytized cells were confounded by changes in focal plane and altered mitochondrial dynamics in expanding hyphal cells. To obtain quantitative results regarding the induced expression of Gdh2-GFP and Put2-GFP within phagosomes, we co-cultured RAW cells with the opsonized reporter strain CFG324 and CFG259 in HBSS for 1 h and analyzed the GFP intensity of both engulfed and external fungal cells. Strains CFG324 and CFG259 are CFG273 (*GDH2*/*GDH2-GFP*) and CFG219 (*PUT2*/*PUT2-GFP*) strains, respectively, engineered to constitutively express RFP under the control of the strong *ADH1* promoter (P_*ADH1*_*-RFP*). The RFP signal was used to locate fungal cells engulfed by macrophages and to normalize the levels of GFP expression. As shown in Fig 4C, the Gdh2-GFP and Put2-GFP signals increased significantly in engulfed fungal cells compared to external cells. The induction of both *GDH2* and *PUT2* is presumably the reflection of limiting glucose availability and release from glucose repression.

**Fig 2:**
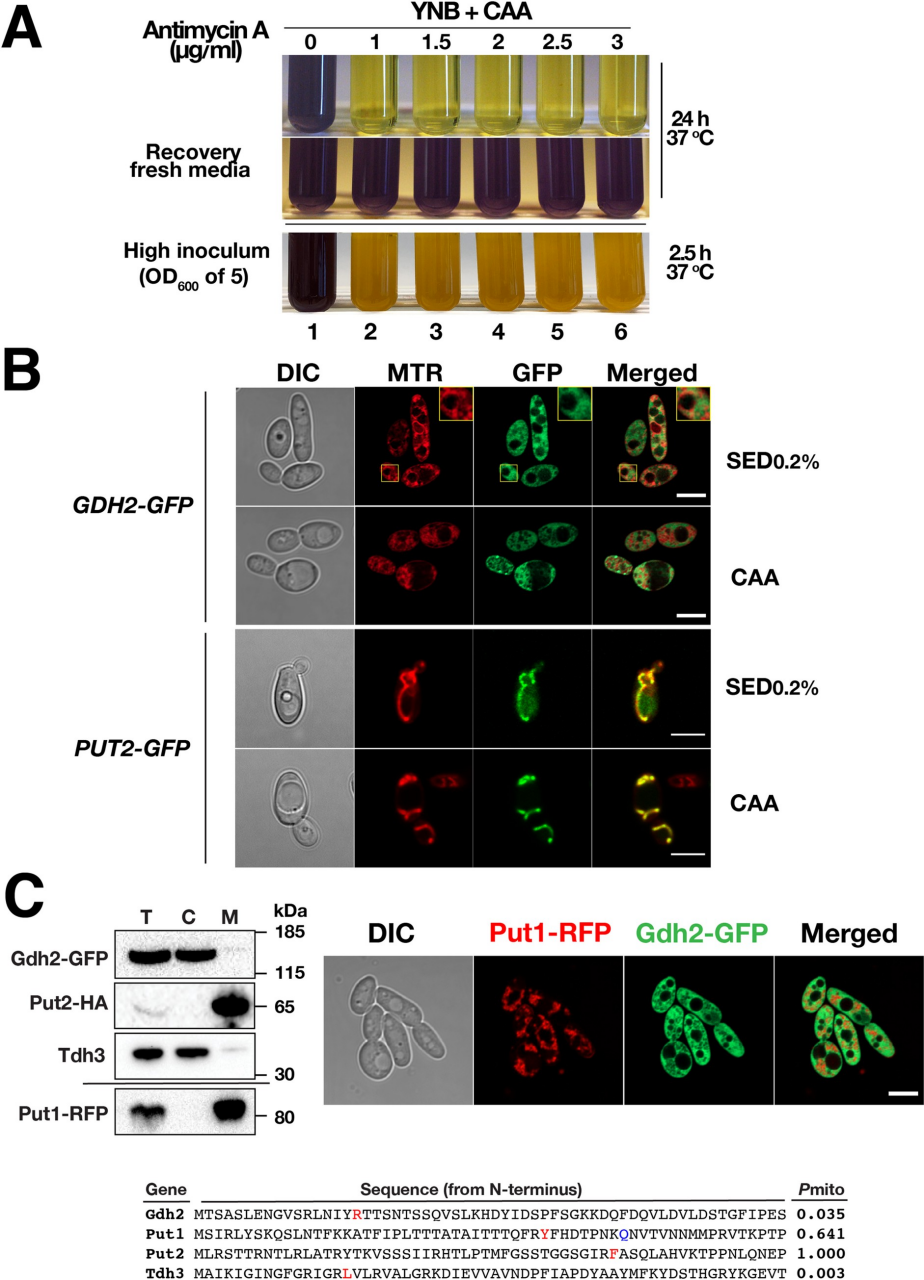
*C*. *albicans* Gdh2 is cytosolic but alkalization is dependent on mitochondrial function. (A) Wildtype cells (SC5314) from overnight YPD cultures were washed and diluted to either OD_600_ ≈ 0.1 (top panel) or ≈ 5 (bottom panel) in liquid YNB+CAA with the indicated concentrations of mitochondrial complex III inhibitor antimycin A. Cultures were grown at 37°C under constant aeration for 24 h and 2.5 h, respectively, and photographed. To assess viability after antimycin A treatment, inhibited cells from 24 h old culture (top panel) were harvested, washed, and resuspended in fresh YNB+CAA media and incubated for 24 h at 37°C (middle panel). Images are representative of at least 3 independent experiments. (B) Strains expressing either Gdh2-GFP (CFG273) or Put2-GFP (CFG219) were grown overnight in YPD, harvested, washed, resuspended in SED (with 0.2% glucose) or YNB + CAA and grown for 24 h at 37°C, and stained with 200 nM MTR prior to imaging by differential interference contrast (DIC) and confocal fluorescence microscopy; the scale bar = 5 μm. The inset in the Gdh2-GFP panels (higher magnification) shows that the GFP and MTR signals do not overlap. (C) Fractionation of extracts from cells expressing Gdh2-GFP and Put2-HA (CFG397 or CFG404) or Gdh2-GFP and Put1-RFP (CFG398 or CFG407) (upper left panels). Cells, pre-grown in YPD, were shifted to YNB+CAA (OD_600_ ≈ 2) and grown for 2 h at 37°C. Cytosolic (C) and mitochondrial (M) fractions from total cell lysate (T) were analyzed by immunoblotting using Put2-HA and Tdh3 (GAPDH) as mitochondrial and cytosolic markers, respectively. Gdh2-GFP colocalizes with Tdh3, whereas Put1-RFP colocalizes with Put2-HA. Blots shown are representative of 3 biological replicates. Confocal microscopy of Gdh2-GFP and Put1-RFP fluorescence confirms that Gdh2-GFP exhibits an extramitochondrial localization (upper right panels); Airyscan-processed images—LSM800 confocal microscope, scale bar = 5 μm. The N-terminal regions of Gdh2, Put1, Put2 and Tdh3 were queried for functioning as a mitochondrial targeting sequence (Mitofates; lower panel). The N-termini of Gdh2 and Tdh3, in contrast to Put1 and Put2, have low probability scores. Predicted cleavage sites for mitochondrial processing peptidase (MPP, red font) and mitochondrial intermediate peptidase (Oct1, blue font).

**Fig 3:**
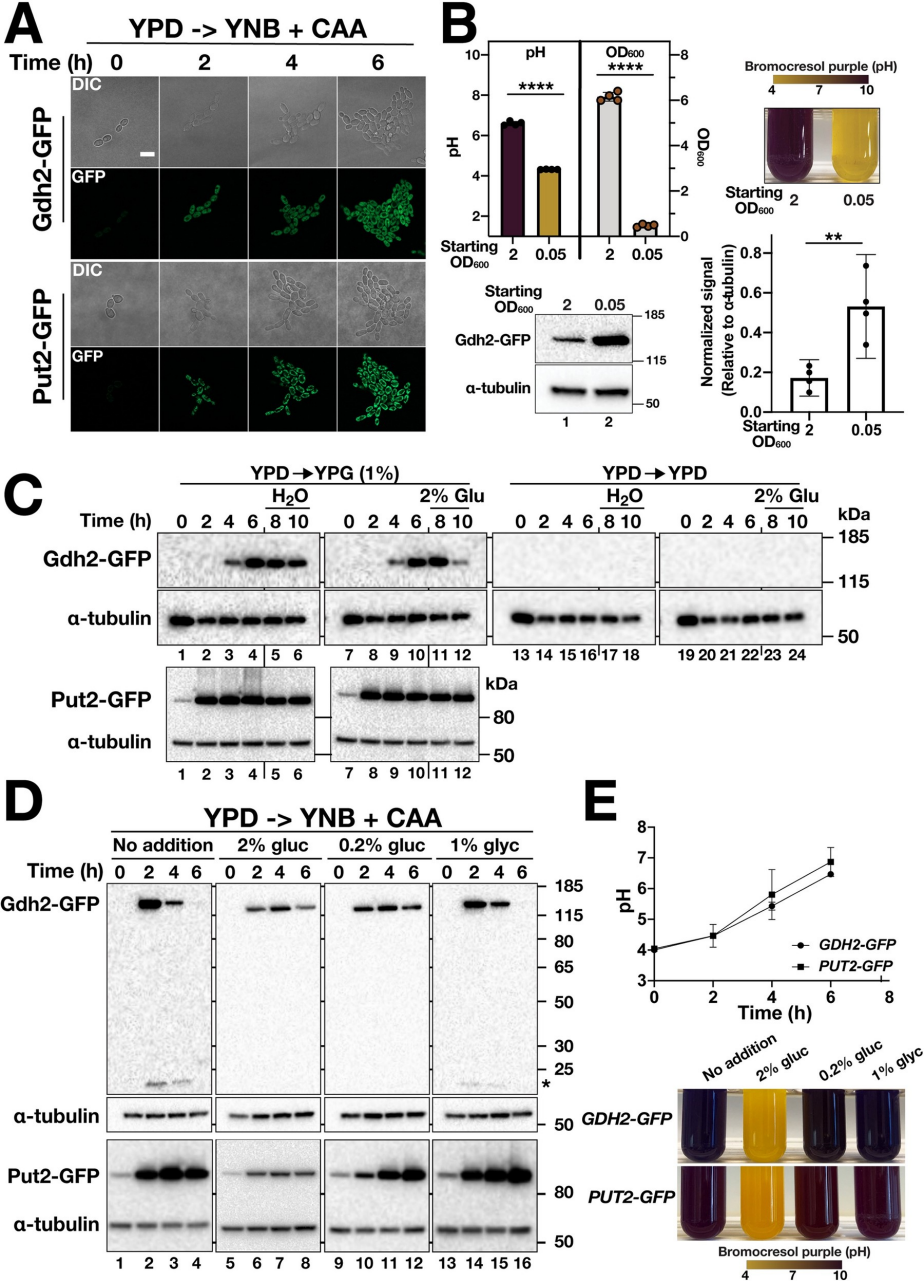
*GDH2* expression is repressed by glucose. (A) Live cell imaging of Gdh2-GFP (CFG273) and Put2-GFP (CFG219) expression in cells shifted from YPD to YNB+CAA. Cells were pre-grown in YPD, transferred to a thin agar slab of YNB+CAA and then observed by confocal microscopy at 37°C. Growth and the intensity of the GFP signals were monitored every hour for 6 h (images at 2 h intervals are shown); Scale bar = 20 μm. (B) Gdh2-GFP expression is cell density-dependent. Washed cells (CFG273) collected from a YPD preculture were inoculated into YNB+CAA with BCP medium at high (≈2) and low (≈0.05) starting OD_600_ and grown for 6 h at 37°C. Samples were collected for pH measurement, OD_600_, and immunoblot analysis. Cultures with high starting cell density reached alkaline pH faster than low starting cell density (top panel, left). Tubes after 6 h of growth between cultures of two starting cell densities are shown for comparison (top panel, right). (Bottom panels) Immunoblot of Gdh2-GFP and α-tubulin (as loading control) in cells from the same 6 h time point and their corresponding quantification. Data shown were derived from 4 biological replicates (Ave. ± CI; ** p ≤ 0.01; **** p ≤ 0.0001). (C) Gdh2-GFP and Put2-GFP expression is sensitive to glucose. (Top panels) Cells (CFG273) grown in YPD were harvested, transferred to YPG (YP + 1% glycerol) or YPD (YP + 2% glucose) at OD_600_ ≈ 2.0, grown for 10 h at 30°C; after 6 h, 2% glucose (lanes 5–6) or ddH_2_O (lanes 11–12) was added, and cultures were grown for an additional 4 h; (Bottom panels) Put2-GFP expressing cells (CFG219) were grown and treated similarly. The levels of Gdh2-GFP and Put2-GFP were determined at 2 h intervals as indicated. (D) Gdh2-GFP (CFG273) and Put2-GFP (CFG219) expression is carbon source dependent. YPD grown cells (OD_600_ ≈ 2.0) were shifted to YNB + CAA alone (lanes 1–4), with 2% glucose (lanes 5–8), 0.2% glucose (lanes 9–12) or 1% glycerol (lanes 13–16) and grown 6 h at 37°C. Extracts were prepared at the times indicated and the levels of Gdh2-GFP, Put2-GFP and α-tubulin (loading control) were assessed by immunoblotting. (E) The pH of liquid cultures of CFG273 (*GDH2-GFP*) and CFG219 (*PUT2-GFP*) grown in YNB+CAA at a starting OD_600_ ≈ 2.0; photograph of tubes after 6 h of growth.

**Fig 4:**
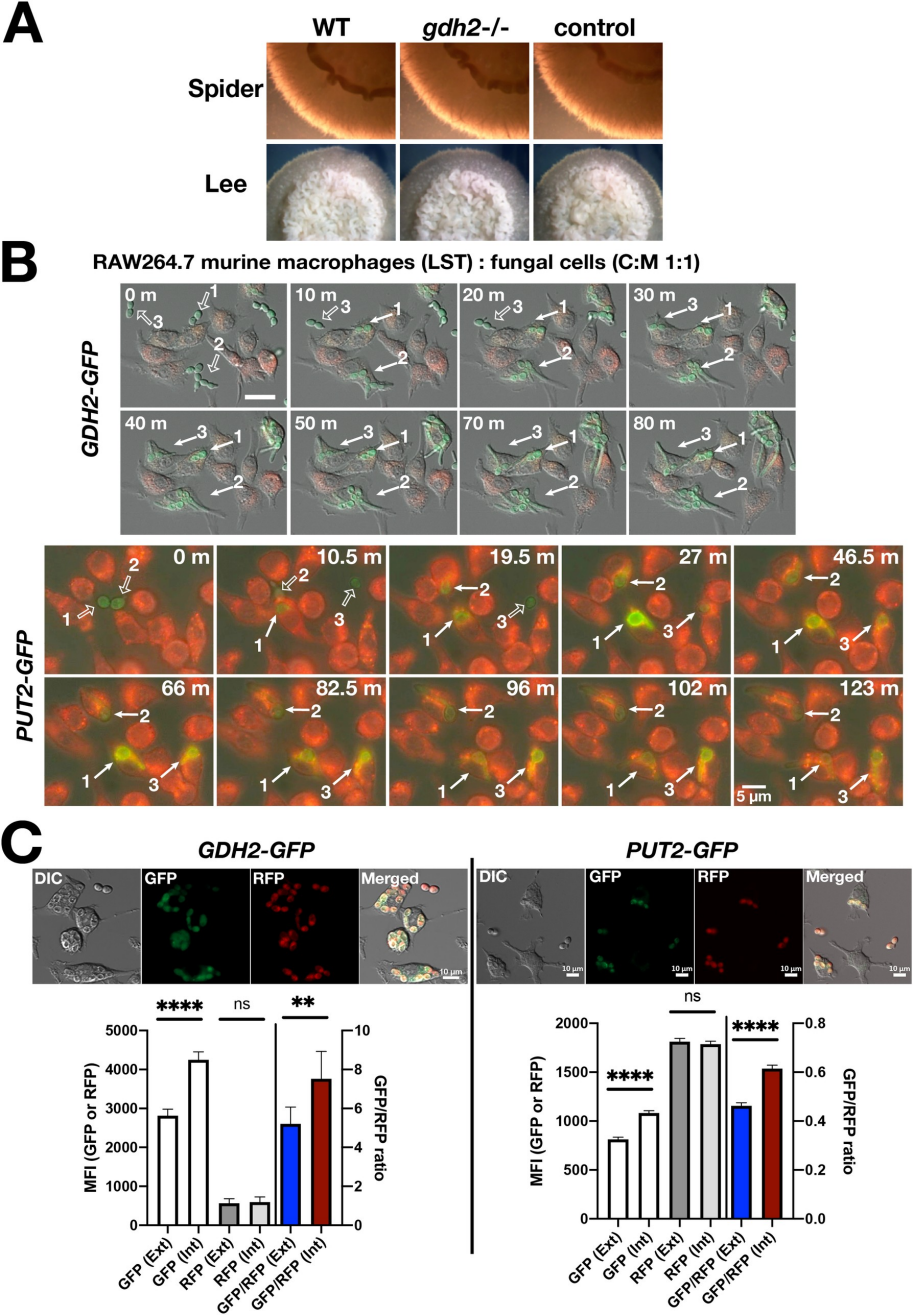
Gdh2 is dispensable for filamentous growth on solid media and is induced in *C*. *albicans* cells phagocytized by murine macrophages. (A) Wildtype (WT, SC5314), *gdh2*-/- (CFG279), and CRISPR control (CFG182) strains, pre-grown in YPD, were washed, resuspended at an OD_600_ ≈ 1 in water, and 5 μl aliquots were spotted on solid Spider and Lee’s media. Representative colonies were photographed 5 days after incubation at 37°C. (B) CFG273 (*GDH2-GFP*) or CFG219 (*PUT2-GFP*) cells were co-cultured in CO_2_-independent medium with RAW264.7 macrophages, pre-stained with Lysotracker Red (LST) at MOI of 1:1 (C:M). The co-cultures were followed by live cell imaging. Micrographs were arbitrarily set at t = 0 min when fungal cells are about to be engulfed by the macrophage (S1A Vid (CFG273) and S1B Vid (CFG219)). In each series, cells are marked prior to (open arrows) and after (closed arrows) being phagocytized. (C) Gdh2 expression in phagocytized fungal cells (CFG324) expressing Gdh2-GFP and RFP (P_*ADH1*_*-RFP*). RAW264.7 macrophages were co-cultured with opsonized CFG324 for 1 h in HBSS prior to measuring GFP and RFP intensities in non- (ext) and phagocytized (int) fungal cells. Results obtained for the strain CFG259 (*PUT2-GFP* P_*ADH1*_*-RFP*) are shown for comparison. Results from at least 3 biological replicates (≥ 100 ext and int cells/replicate) are shown (Ave. ± CI; ****p ≤ 0.0001, **p ≤ 0.01 and no significant difference (ns) by Student t-test). Scale bar = 10 μm.

## Supporting information

S1 Fig CRISPR/Cas9-mediated gene inactivation of *GDH2* and construction of a *GDH2-GFP* reporter strain. (A) A purified *Kpn*I/*Sac*I fragment from pFS108, harboring *GDH2*-specific sgRNA, and PCR generated repair template (RT) were introduced into wildtype strain SC5314 by electroporation. Nou^R^ transformants were pre-screened in YNB+Arg medium containing the pH indicator bromocresol purple; the initial pH was 4.0. Three Nou^R^ colonies were picked for further analysis. Clones #1 and #2 grew poorly and were unable to alkalinize the media; clone #3 grew and alkalinized the media. (B) Genomic DNA, isolated from the three clones, was used as template for PCR amplification of the targeted *GDH2* locus; ddH_2_O was used as negative control. Restriction of the amplified ≈900 bp fragment by *Xho*I is diagnostic for successful mutagenesis (primers p5/p6; S2 Table). Strains, clone #1 (CFG277) and clone #2 (CFG278) carry inactivated *gdh2*-/- alleles. (C) *GDH2* is not essential but required for robust growth on glutamate or proline as sole nitrogen source. Five microliters of serially diluted wildtype (SC5314), *gdh2*-/- NAT^R^ (CFG277), *gdh2*-/- NAT^S^ (CFG279), and control (CFG182) cells were spotted on yeast peptone (YP), synthetic glutamate (SE) and synthetic proline (SP) media containing either 2% glucose (YPD, SED, SPD) or 1% glycerol (YPG, SEG, SPG) as carbon source. The plates were incubated for 48 h at 30°C and photographed. (D) Fresh colonies of SC5314 (PLC005; WT), CFG279 (*gdh2*-/-), CASJ041 (*cph1*-/- *efg1*-/-) and CFG352 (*cph1*-/- *efg1*-/- *gdh2*-/-) were individually resuspended in YNB+CAA medium and incubated for 24 h at 37°C. (E) The insertion of GFP in strain CFG273 (*GDH2-GFP*) was verified by PCR, the expected 1695 bp fragment was amplified using primers (p24/p25; S2 Table); strain CAI4 served as untagged control (middle left panel). CFG273 was transformed with the CRISPR/Cas9 cassette to inactivate *GDH2*. Putative *gdh2*-/- clones were identified as described and verified by PCR-RD (p13/p6; S2 Table) resulting in strain CFG400. Strains CFG273 (*GDH2/GDH2-GFP*) and CFG400 (*gdh2*/*gdh2-GFP*) were grown at a starting OD_600_ ≈ 2 in SE medium with 0.2% glucose and 1 mM proline (SED 0.2%+Pro) for 2 h. In contrast to CFG273, strain CFG400 failed to express Gdh2-GFP as assessed by immunoblot (middle right panel) and microscopy (lower panels; Scale bar = 5 μm), demonstrating the specificity of CRISPR/Cas9.
(TIF)

S6 Fig Gdh2 expression is repressed by glucose. (A) Cells expressing *GDH2-GFP* (CFG273) were collected from YPD overnight cultures, washed, and then diluted in liquid YNB+CAA with or without the indicated concentrations of glucose or glycerol at OD_600_ ≈ 2.0 and then incubated under aeration at 37°C. Cells were harvested at the indicated time points and then immediately washed with ddH_2_O for microscopic examination of Gdh2-GFP expression. Representative images of cells from each condition with their relative expression of Gdh2-GFP are shown. Results obtained for the strain CFG219 (*PUT2-GFP*) are shown for comparison. Scale bar = 10 μm. (B) (Left) Cells collected from no addition (T = 4h) were stained with MitoTracker Deep Red (MTR; 200 nM) and then observed by confocal microscopy (LSM800) using the Airyscan detector. The Gdh2-GFP and MTR signals are mutually exclusive whereas the Put2-GFP colocalizes with MTR; Scale bar = 2 μm. (Right) Quantification of mean fluorescence intensity (MFI) from at least 3 biological replicates per condition (≥150 cells/replicate) are shown.
(TIF)

S1 Vid A) Gdh2-GFP (CFG273) is induced upon phagocytosis by RAW264.7 macrophages (Fig 4B). B) Put2-GFP (CFG219) is induced upon phagocytosis by RAW264.7 macrophages (Fig 4B).
(ZIP)

S2 Vid A) Gdh2-GFP (CFG273) is induced upon phagocytosis by primary murine BMDM. B) Put2-GFP (CFG219) is induced upon phagocytosis by primary murine BMDM.
(ZIP)
