# Correction: Correction: Glutamate dehydrogenase (Gdh2)-dependent alkalization is dispensable for escape from macrophages and virulence of *Candida albicans*

**DOI:** 10.1371/journal.ppat.1010008

**Published:** 2021-10-19

**Authors:** The PLOS Pathogens Staff

The S1 Fig is incorrect. The correct version appears below. The publisher apologizes for the error.

## Supporting information

S1 Fig CRISPR/Cas9-mediated gene inactivation of *GDH2* and construction of a *GDH2-GFP* reporter strain. (A) A purified *Kpn*I/*Sac*I fragment from pFS108, harboring *GDH2*-specific sgRNA, and PCR generated repair template (RT) were introduced into wildtype strain SC5314 by electroporation. Nou^R^ transformants were pre-screened in YNB+Arg medium containing the pH indicator bromocresol purple; the initial pH was 4.0. Three Nou^R^ colonies were picked for further analysis. Clones #1 and #2 grew poorly and were unable to alkalinize the media; clone #3 grew and alkalinized the media. (B) Genomic DNA, isolated from the three clones, was used as template for PCR amplification of the targeted *GDH2* locus; ddH_2_O was used as negative control. Restriction of the amplified ≈900 bp fragment by *Xho*I is diagnostic for successful mutagenesis (primers p5/p6; S2 Table). Strains, clone #1 (CFG277) and clone #2 (CFG278), carry inactivated *gdh2-/-* alleles. (C) *GDH2* is not essential but required for robust growth on glutamate or proline as sole nitrogen source. Five microliters of serially diluted wildtype (SC5314), *gdh2-/-* NAT^R^ (CFG277), *gdh2-/-* NAT^S^ (CFG279), and control (CFG182) cells were spotted on yeast peptone (YP), synthetic glutamate (SE) and synthetic proline (SP) media containing either 2% glucose (YPD, SED, SPD) or 1% glycerol (YPG, SEG, SPG) as carbon source. The plates were incubated for 48 h at 30 °C and photographed. (D) Fresh colonies of SC5314 (PLC005; WT), CFG279 (*gdh2-/-*), CASJ041 (*cph1-/- efg1-/-*) and CFG352 (*cph1-/- efg1-/- gdh2-/-*) were individually resuspended in YNB+CAA medium and incubated for 24 h at 37 °C. (E) The insertion of GFP in strain CFG273 (*GDH2-GFP*) was verified by PCR, the expected 1695 bp fragment was amplified using primers (p24/p25; S2 Table); strain CAI4 served as untagged control (middle left panel). CFG273 was transformed with the CRISPR/Cas9 cassette to inactivate *GDH2*. Putative *gdh2-/-* clones were identified as described and verified by PCR-RD (p13/p6; S2 Table) resulting in strain CFG400. Strains CFG273 (*GDH2/GDH2-GFP*) and CFG400 (*gdh2/gdh2-GFP*) were grown at a starting OD_600_ ≈ 2 in SE medium with 0.2% glucose and 1 mM proline (SED 0.2%+Pro) for 2 h. In contrast to CFG273, strain CFG400 failed to express Gdh2-GFP as assessed by immunoblot (middle right panel) and microscopy (lower panels; Scale bar = 5 μm), demonstrating the specificity of CRISPR/Cas9. https://doi.org/10.1371/journal.ppat.1009877.s001.
(TIF)

## References

[1] Silao FGS, Ryman K, Jiang T, Ward M, Hansmann N, Molenaar C, et al. (2020) Glutamate dehydrogenase (Gdh2)-dependent alkalization is dispensable for escape from macrophages and virulence of *Candida albicans*. PLoS Pathog 16(9): e1008328. doi: 10.1371/journal.ppat.1008328 32936835 PMC7521896

[2] Silao FGS, Ryman K, Jiang T, Ward M, Hansmann N, Molenaar C, et al. (2021) Correction: Glutamate dehydrogenase (Gdh2)-dependent alkalization is dispensable for escape from macrophages and virulence of *Candida albicans*. PLoS Pathog 17(8): e1009877. doi: 10.1371/journal.ppat.1009877 34460867 PMC8405231

