# Supplementary figures and images for: Glutamate dehydrogenase (Gdh2)-dependent alkalization is dispensable for escape from macrophages and virulence of Candida albicans
Source: PLoS Pathog. 2020 Sep 16;16(9):e1008328. doi: 10.1371/journal.ppat.1008328 (PMC7521896; doi:10.1371/journal.ppat.1008328)

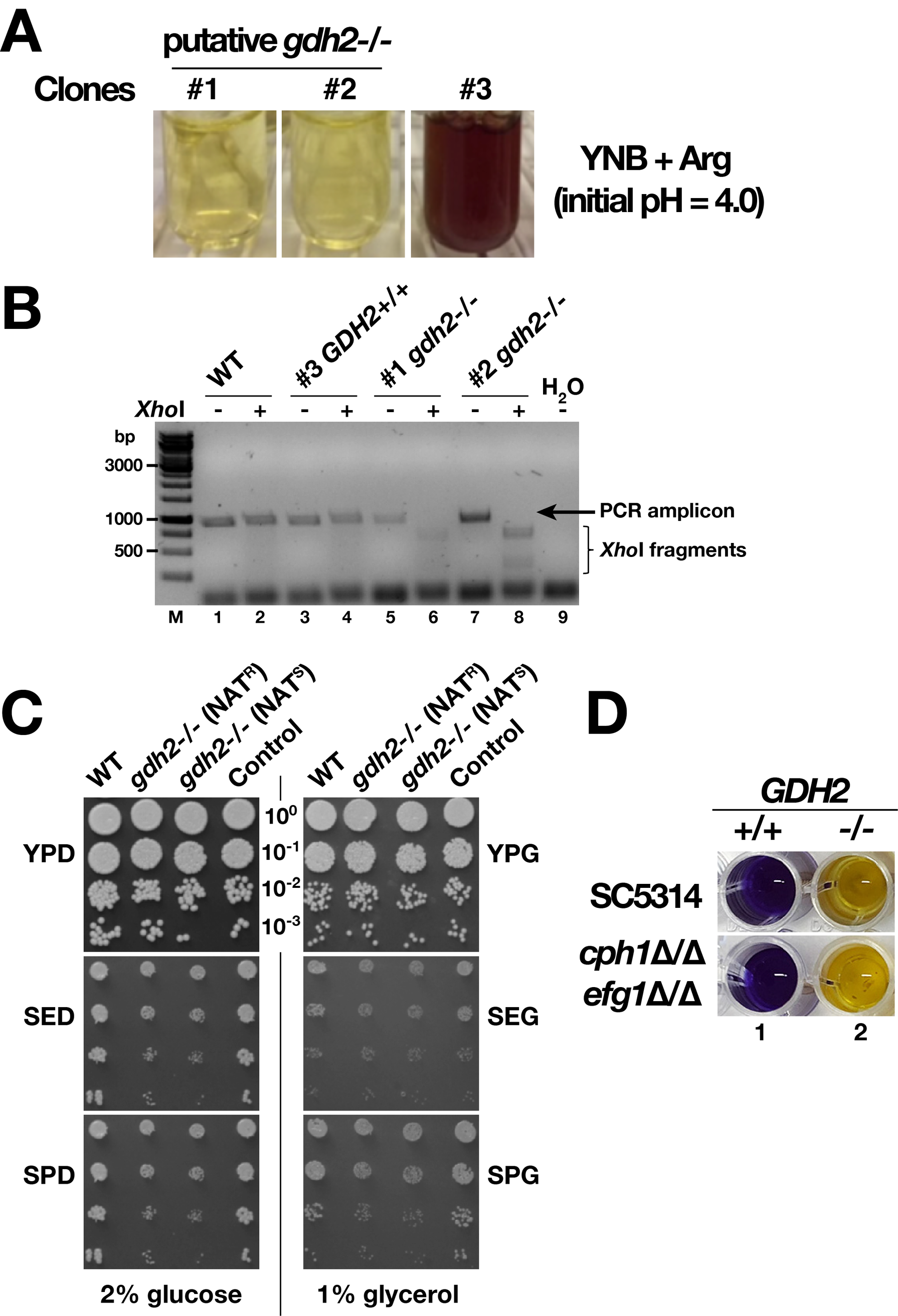

Supplement: S1 Fig — (A) A purified KpnI/SacI fragment from pFS108, harboring GDH2-specific sgRNA, and PCR generated repair template (RT) were introduced into wildtype strain SC5314 by electroporation. NouR transformants were pre-screened in YNB+Arg medium containing the pH indicator bromocresol purple; the initial pH was 4.0. Three NouR colonies were picked for further analysis. Clones #1 and #2 grew poorly and were unable to alkalinize the media; clone #3 grew and alkalinized the media. (B) Genomic DNA, isolated from the three clones, was used as template for PCR amplification of the targeted GDH2 locus; ddH2O was used as negative control. Restriction of the amplified ≈900 bp fragment by XhoI is diagnostic for successful mutagenesis. Strains, clone #1 (CFG277) and clone #2 (CFG278) carry inactivated gdh2-/- alleles. (C) GDH2 is not essential but required for robust growth on glutamate or proline as sole nitrogen source. Five microliters of serially diluted wildtype (SC5314), gdh2-/- NATR (CFG277), gdh2-/- NATS (CFG279), and control (CFG182) cells were spotted on yeast peptone (YP), synthetic glutamate (SE) and synthetic proline (SP) media containing either 2% glucose (D) or 1% glycerol (G) as carbon source. The plates were incubated for 48 h at 30°C and photographed. (D) A colony from fresh YPD plates were directly inoculated onto a single well of a 96-well plate containing YNB+CAA medium and then incubated for 24 h at 37°C. (TIF) [file ppat.1008328.s001.tif]

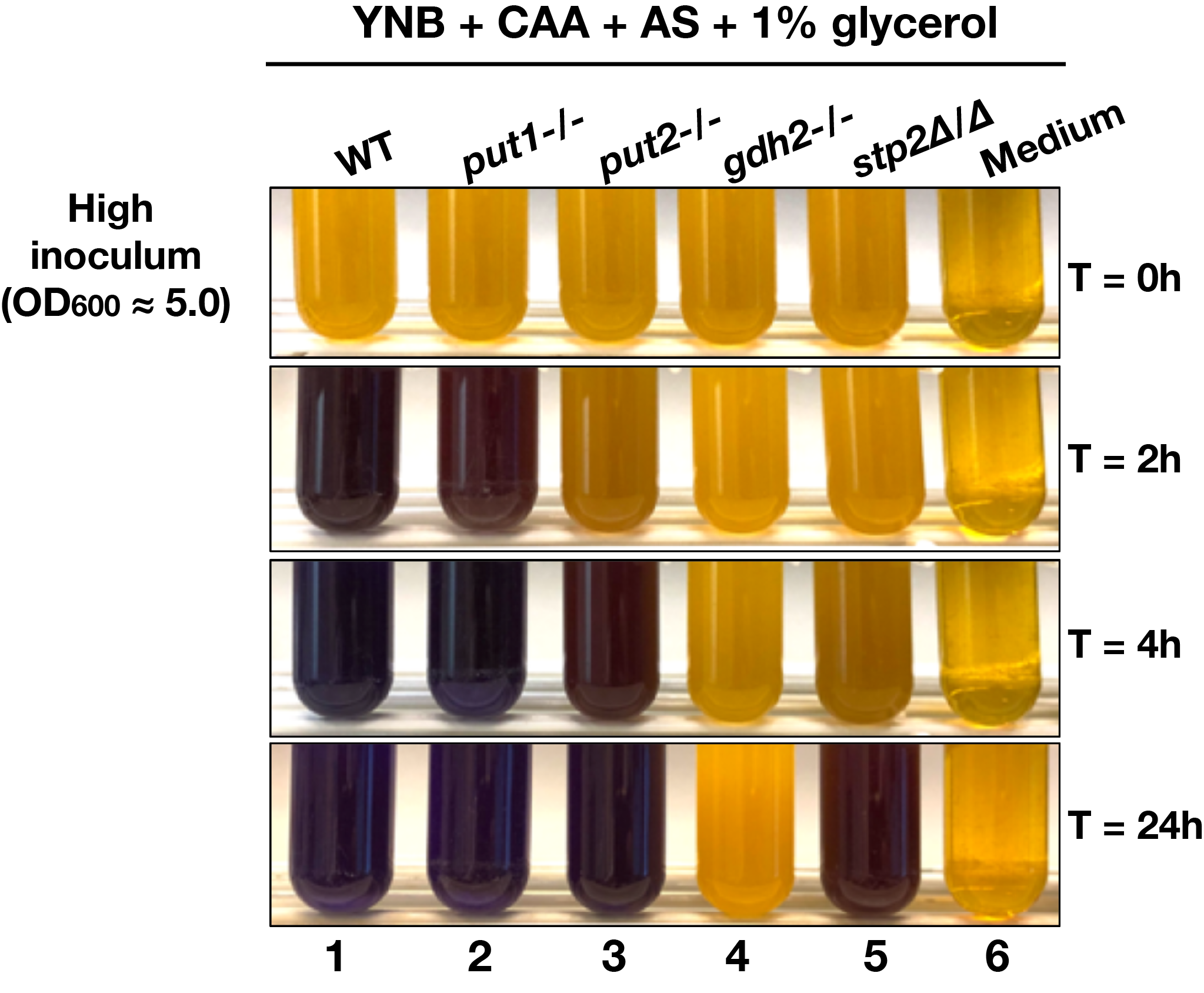

Supplement: S2 Fig — Cells of the indicated strains were collected from YPD overnight culture, washed, and then diluted to OD600 ≈ 5.0 in YNB+CAA medium supplemented with 38 mM ammonium sulfate (AS) and 1% glycerol. Tubes were incubated at 37°C and then photographed at the indicated times. Only gdh2-/- mutant failed to alkalinize the medium. Strains used: WT (SC5314), put1-/- (CFG154), put2-/- (CFG318), gdh2-/- (CFG279) and stp2Δ/Δ (SVC17). (TIF) [file ppat.1008328.s002.tif]

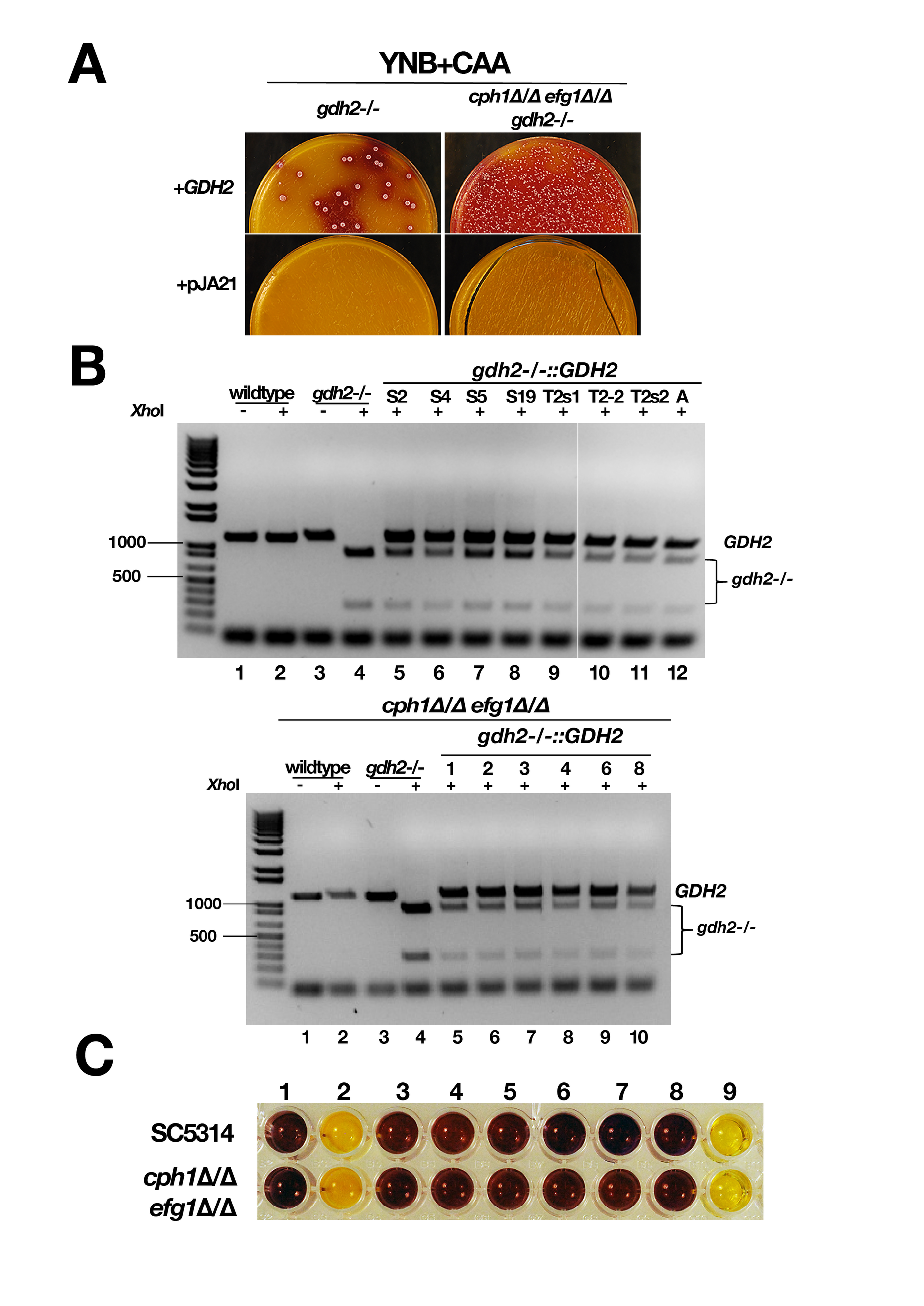

Supplement: S3 Fig — (A) Strains CFG279 (gdh2-/-) and CFG354 (cph1Δ/Δ efg1Δ/Δ gdh2-/-) were transformed with wildtype GDH2 gene fragment that encompasses the mutated region of GDH2. An unrelated gene fragment (pJA21, PADH1-RFP-caSAT1) was also used to transform the same strains as control. Transformants were selected on YNB+CAA with BCP and 2% agar (pH = 4.0) as control. Transformation plate images taken 2-3 days after incubation at 37°C showing alkalization positive transformants. (B) Purified colonies from (A) were verified by PCR followed by XhoI restriction digest (RD). Heterozygotes (gdh2-/GDH2) were identified by the presence of both mutated and wildtype GDH2 alleles. PCR-RD verification of GDH2/gdh2- (Top) and cph1Δ/Δ efg1Δ/Δ GDH2/gdh2- (Bottom) reconstituted strains. Clones S2, S4, S5, and S19 (Top) and clones 1 and 2 (Bottom) were obtained from a separate transformation experiment were following electroporation, cells were directly recovered and enriched in liquid YNB+CAA for 24 h at 37°C prior to plating on YPD agar for single colonies. (C) All strains were verified again in their capacity to grow and alkalinize the YNB+CAA medium by directly inoculating purified colonies into each well containing medium and then grown statically at 37°C for 24 h. Reconstituted strains shown were randomly selected from the PCR-RD positive clones. Wells in the SC5314 lane: 1 (PLC005), 2 (CFG279), 3 (CFG355; Clone S5), 4 (CFG356; Clone S19), 5 (CFG357; Clone T2s1), 6 (CFG358; Clone T2-2), 7 (gdh2-/GDH2; Clone T2s2), 8 (GDH2/gdh2-; Clone A), and 9 (Medium); wells in the cph1Δ/Δ efg1Δ/Δ lane: 1 (CASJ041), 2 (CFG354), 3 (CFG359; Clone 1), 4 (CFG360; Clone 2), 5 (CFG361; Clone 3), 6 (CFG362; Clone 4), 7 (cph1Δ/Δ efg1Δ/Δ GDH2/gdh2-; Clone 6), 8 (cph1Δ/Δ efg1Δ/Δ GDH2/gdh2-; Clone 8), and 9 (Medium). Reconstituted strains containing the “CFG-” code were stored at -80°C as glycerol stocks and were listed in the strains list. (TIF) [file ppat.1008328.s003.tif]

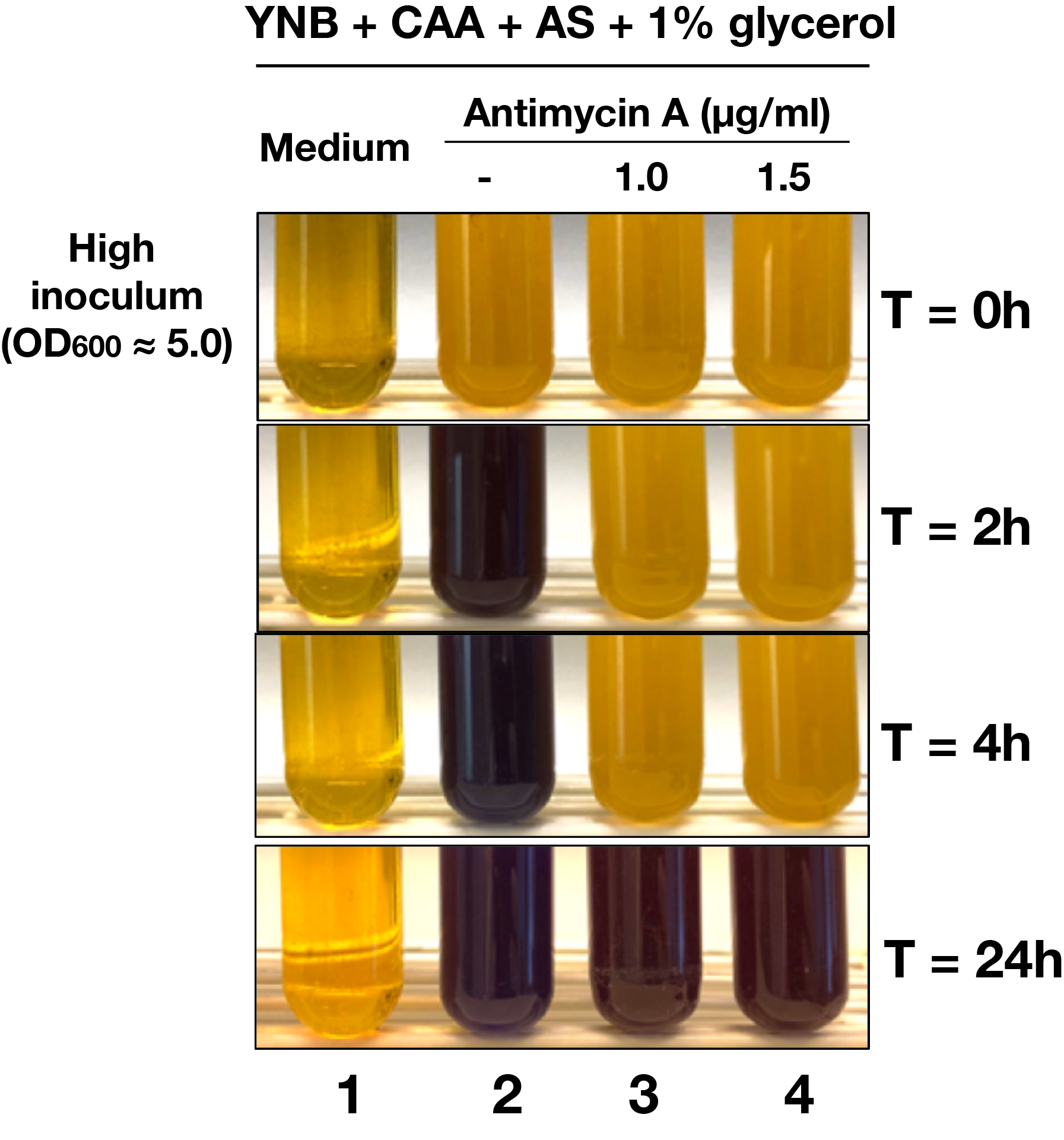

Supplement: S4 Fig — Wildtype cells (SC5314) collected from YPD overnight cultures were washed and then diluted to OD600 ≈ 5 in liquid YNB+CAA medium supplemented with 38 mM ammonium sulfate (AS) and 1% glycerol with the indicated concentrations of mitochondrial complex III inhibitor antimycin A. Cultures were incubated continuously at 37°C under constant aeration and then photographed at the indicated time points. Images are representative of at least 3 independent experiments. For control (-), equal amount of ethanol carrier as that of 1.5 μg/ml of antimycin A was added to the tube. (TIF) [file ppat.1008328.s004.tif]

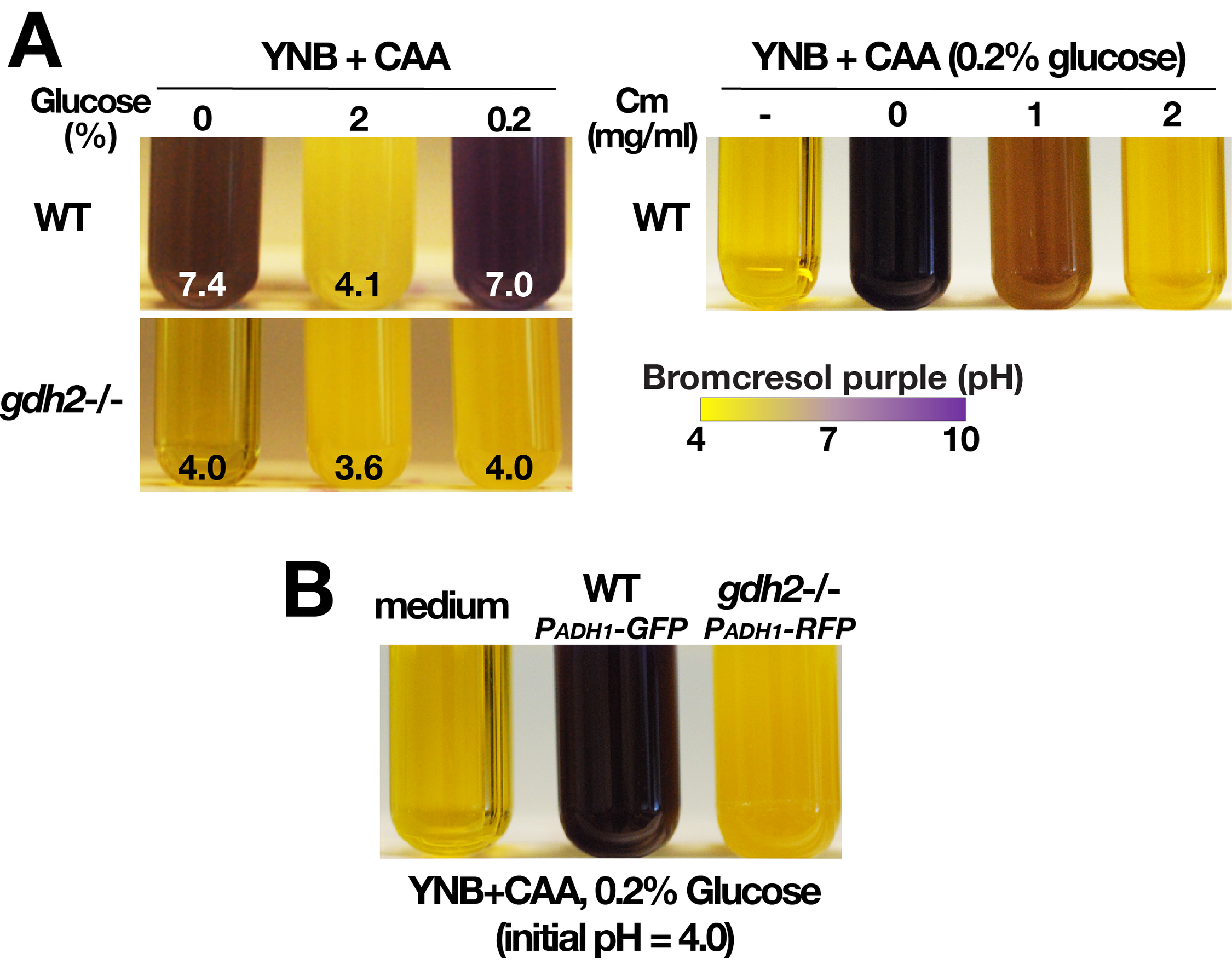

Supplement: S5 Fig — (A) Gdh2-dependent alkalization is sensitive to glucose (Left panel). YPD grown wildtype (WT, SC5314) and gdh2-/- (CFG279) cells were collected, washed, and diluted to an OD600 ≈ 0.05 in YNB+CAA with 0, 2 or 0.2% glucose as indicated. The cultures were grown under vigorous agitation at 37°C for 16 h and the pH was measured (the initial pH was 4.0; the values indicated are the average of three replicate cultures). Alkalization is linked to mitochondrial function (Right panel). Wildtype cells (SC5314) from overnight YPD cultures were washed and diluted to OD600 ≈ 0.1 in liquid YNB+CAA (0.2% glucose) with the indicated concentrations of mitochondrial translation inhibitor chloramphenicol. Cultures were grown at 37°C under vigorous agitation for 16 h. (B) Phenotypic validation of the reporter strains used in macrophage co-cultures. Growth of wildtype (WT; PADH1-GFP-caSAT1; SCADH1G4A) and gdh2-/- (PADH1-RFP-caSAT1, CFG275) cells in YNB+CAA supplemented with 0.2% glucose. Cultures were grown for 16 h at 37°C. (TIF) [file ppat.1008328.s005.tif]

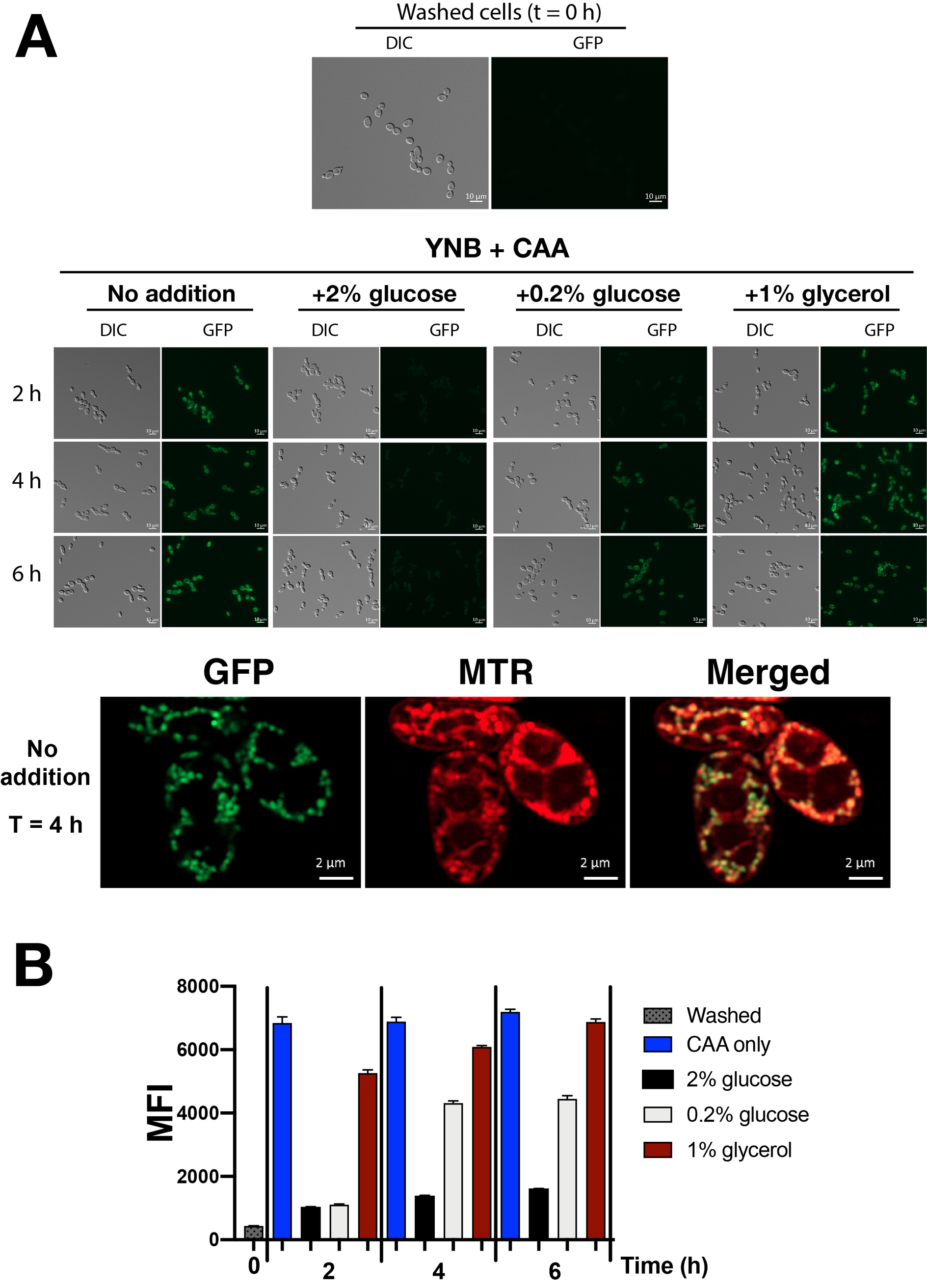

Supplement: S6 Fig — (A) Cells expressing Gdh2-GFP (CFG273) were collected from YPD overnight cultures, washed, and then diluted in liquid YNB+CAA with or without the indicated concentrations of glucose or glycerol at OD600 ≈ 2.0 and then incubated under aeration at 37°C. Cells were harvested at the indicated time points and then immediately washed with ddH2O for microscopic examination of Gdh2-GFP expression. Representative images of cells from each condition with their relative expression of Gdh2-GFP are shown. (Bottom panel) Cells collected from one of the conditions (i.e., no addition; T = 4h) were stained with MitoTracker Deep Red (MTR; 200 nM) and then observed by confocal microscopy (LSM800) using the Airyscan detector. The Gdh2-GFP signal colocalizes with MTR. (B) Quantification of mean fluorescence intensity (MFI) from 3 biological replicates per condition (≥150 cells/replicate) are shown. (TIF) [file ppat.1008328.s006.tif]

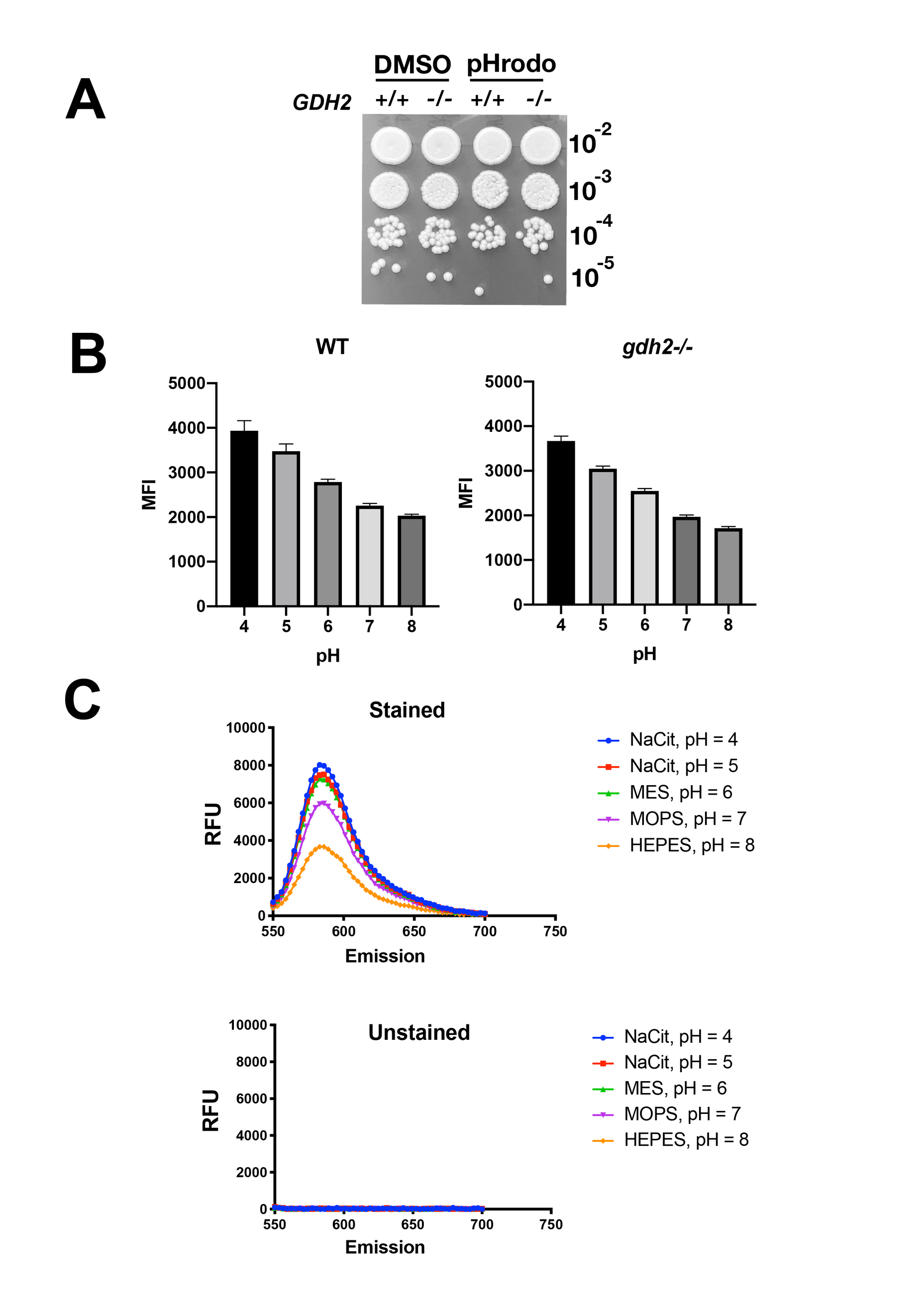

Supplement: S7 Fig — Mid log phase cells from YPD culture were harvested, washed, and then stained with pHrodo (or DMSO as control) in NaHCO3 buffer as outlined (Methodology). (A) Viability assessment of cells stained with the dye and then opsonized prior to infection. Stained cells were serially diluted in PBS and then an aliquot (5 μl) spotted on YPD. Photographs taken after 48 h of growth at 30°C. (B) A 2-μl aliquot of stained cells were added to imaging dish containing 2.5 ml of the buffer, equilibrated for at least 5 min, and then cells were imaged at 37°C. Quantification of mean fluorescence intensity (MFI) from 3 biological replicates per condition (≥150 cells/replicate) are shown. (Ave. ± CI; **** p £ 0.0001 by one-way ANOVA). (C) Ten-μl aliquots of samples were added to 190 μl of buffer and analyzed for emission using Enspire reader with excitation of 532 nm. (TIF) [file ppat.1008328.s007.tif]

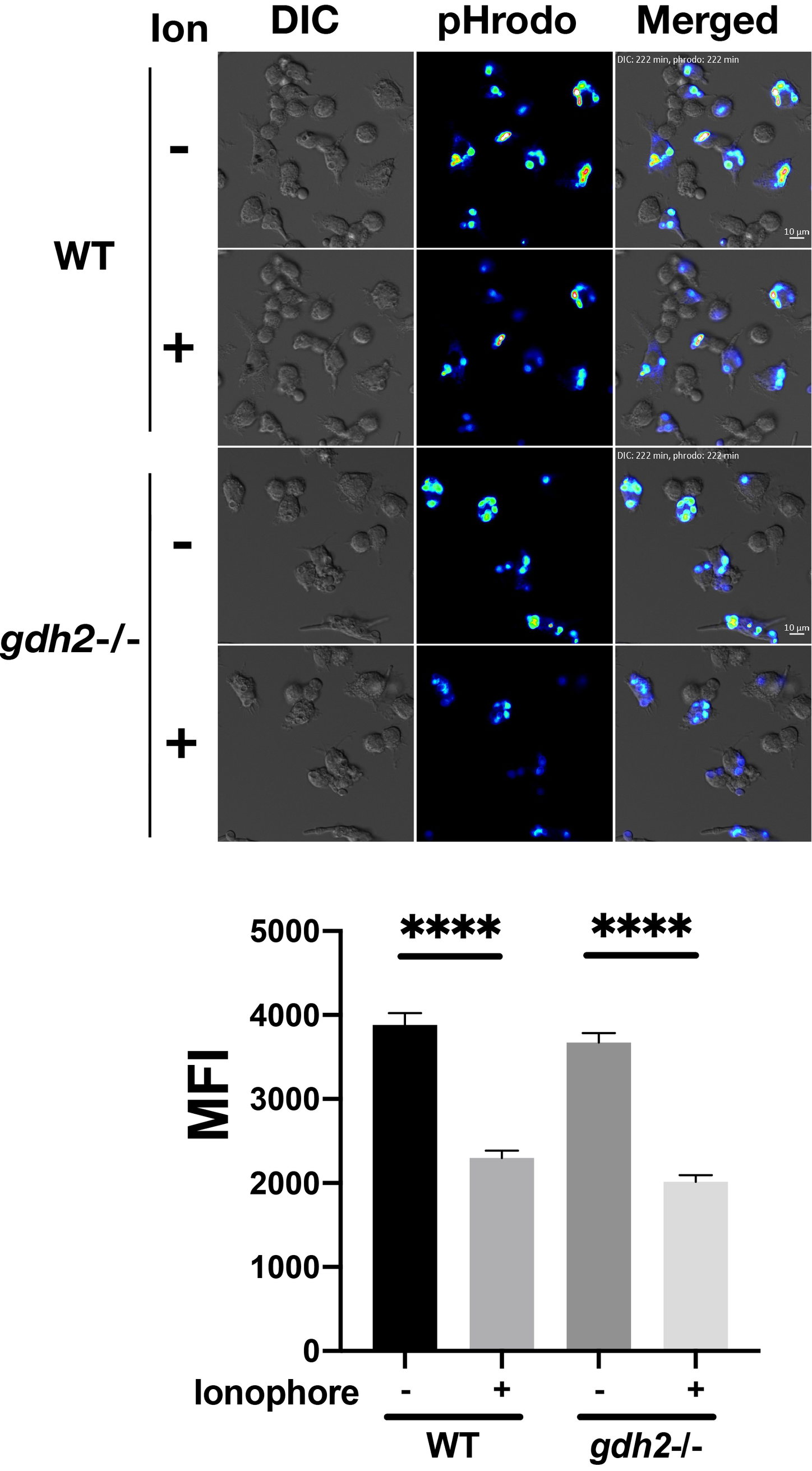

Supplement: S8 Fig — (Top) pHrodo-stained wildtype (PLC005) and gdh2-/- (CFG279) cells were co-cultured with macrophage for around 4 h and then treated with 10 μM of both monensin and nigericin (i.e., Ionophores, Ion) for 5 min to dissipate proton gradients in acidified compartments. Co-cultures were photographed before and after addition of ionophores. (Bottom) Quantification of phagosome intensities before and after addition of ionophores. At least 100 phagosomes/replicate were analyzed (Ave. ± CI; **** p £ 0.0001 by Student t-test). (TIF) [file ppat.1008328.s008.tif]
