# Supplementary material for: Glutamate dehydrogenase (Gdh2)-dependent alkalization is dispensable for escape from macrophages and virulence of Candida albicans
Source: PLoS Pathog. 2020 Sep 16;16(9):e1008328. doi: 10.1371/journal.ppat.1008328 (PMC7521896; doi:10.1371/journal.ppat.1008328)
Supplement: S1 Table — (DOCX) [file ppat.1008328.s009.docx]

| **S1 Table Strains used in this study** | | | | |  |
| --- | --- | --- | --- | --- | --- |
| Strain | | Genotype | Reference | | |
| CAI4-derived strains | | |  | | |
| CAI4 | *ura3Δ::imm434/ura3Δ::imm434* | | [48] |  |  |
| CFG273 | *ura3Δ::imm434/ura3Δ::imm434 GDH2/GDH2-GFP-URA3* | | This work |  |  |
| CFG321 | *ura3Δ::imm434/ura3Δ::imm434 GDH2/GDH2-GFP-URA3 ADH1/adh1::P_ADH1_-RFP-caSAT1* | | This work |  |  |
| CFG324 | *ura3Δ::imm434/ura3Δ::imm434 GDH2/GDH2-GFP-URA3 ADH1/adh1::P_ADH1_-RFP-FRT (pop-out of CFG321)* | | This work |  |  |
| SC5314-derived strains | | |  | | |
| PLC005 | | SC5314; *Prototrophic wild type* |  | | |
| CASJ041 | | *cph1Δ::FRT/cph1Δ::FRT efg1Δ::FRT/efg1Δ::FRT* | [19] | | |
| CFG154 | | *NEUT5L::FRT put1-/-* | [15] | | |
| CFG159 | | *NEUT5L::FRT put1- /- ENO1/eno1::P_ENO1_-CC9-pFS083 put2- /-* | [15] | | |
| CFG318 | | *NEUT5L::FRT put2- /-* | [15] | | |
| CFG182 | | *NEUT5L::pV1524* | [15] | | |
| CFG246 | | *ENO1/eno1::P_ENO1_-CC9-pFS039 dur1,2-/-* | [15] | | |
| CFG275 | | *NEUT5L::FRT gdh2-/- ADH1/adh1::P_ADH1_-RFP- caSAT1* | This work | | |
| CFG277 | | *NEUT5L::P_ENO1_-CC9-pFS108 gdh2-/- (Clone 1)* | This work | | |
| CFG278 | | *NEUT5L::P_ENO1_-CC9-pFS108 gdh2-/- (Clone 2)* | This work | | |
| CFG279 | | *NEUT5L::FRT gdh2-/- (from CFG277)* | This work | | |
| CFG281 | | *NEUT5L::FRT gdh2-/- (from CFG278)* | This work | | |
| CFG283 | | *NEUT5L::P_ENO1_-CC9-pFS108 GDH2+/+* | This work | | |
| CFG346 | | *cph1Δ::FRT/cph1Δ::FRT efg1Δ::FRT/efg1Δ::FRT*  *NEUT5L::P_ENO1_-CC9-pFS108 gdh2-/- (Clone K3)* | This work | | |
| CFG347 | | *cph1Δ::FRT/cph1Δ::FRT efg1Δ::FRT/efg1Δ::FRT*  *NEUT5L::P_ENO1_-CC9-pFS108 GDH2+/+ (Clone 32)* | This work | | |
| CFG352 | | *cph1Δ::FRT/cph1Δ::FRT efg1Δ::FRT/efg1Δ::FRT*  *NEUT5L::FRT gdh2-/- (Clone 2 from strain CFG346)* | This work | | |
| CFG354 | | *cph1Δ::FRT/cph1Δ::FRT efg1Δ::FRT/efg1Δ::FRT*  *NEUT5L::FRT gdh2-/- (Clone 6 from strain CFG346)* | This work | | |
| CFG355 | | *NEUT5L::FRT GDH2/gdh2- (Clone S5)* | This work | | |
| CFG356 | | *NEUT5L::FRT GDH2/gdh2- (Clone S19)* | This work | | |
| CFG357 | | *NEUT5L::FRT GDH2/gdh2-(Clone T2S1)* | This work | | |
| CFG358 | | *NEUT5L::FRT GDH2/gdh2-(Clone T2#2)* | This work | | |
| CFG359 | | *cph1Δ::FRT/cph1Δ::FRT efg1Δ::FRT/efg1Δ::FRT*  *NEUT5L::FRT GDH2/gdh2-(Clone C1)* | This work | | |
| CFG360 | | *cph1Δ::FRT/cph1Δ::FRT efg1Δ::FRT/efg1Δ::FRT*  *NEUT5L::FRT GDH2/gdh2- (Clone C10)* | This work | | |
| CFG361 | | *cph1Δ::FRT/cph1Δ::FRT efg1Δ::FRT/efg1Δ::FRT*  *NEUT5L::FRT GDH2/gdh2- (Clone T2C#1)* | This work | | |
| CFG362 | | *cph1Δ::FRT/cph1Δ::FRT efg1Δ::FRT/efg1Δ::FRT*  *NEUT5L::FRT GDH2/gdh2- (Clone T2C#2)* | This work | | |
| SCADH1G4A | | *ADH1/adh1::P_ADH1_-GFP-caSAT1* | [23] | | |
| SVC17 | | *stp2Δ::FRT/stp2Δ::FRT* | [13] | | |
