# Supplementary material for: Glutamate dehydrogenase (Gdh2)-dependent alkalization is dispensable for escape from macrophages and virulence of Candida albicans
Source: PLoS Pathog. 2020 Sep 16;16(9):e1008328. doi: 10.1371/journal.ppat.1008328 (PMC7521896; doi:10.1371/journal.ppat.1008328)
Supplement: S2 Table — (DOCX) [file ppat.1008328.s010.docx]

| **S2 Table Primers used in this study** | | | |
| --- | --- | --- | --- |
| **p#** | **Primer Name** | **Sequence** | **Reference** |
| - 1 | - sgRNA-GDH2T | atttgTACATTGACTCCCCCTTTAGg | This study |
| - 2 | - sgRNA-GDH2B | - aaaacCTAAAGGGGGAGTCAATGTAc | - This study |
| - 3 | - RT-GDH2Top | - GTTTAAACATTTACAGAACCACATCAAACACTTCATCCCAAGTTAGTTTGAAACACGACTAActcgagTAAttTAGGG | - This study |
| - 4 | - RT-GDH2Bot | - CAGGGATAAAACCAGTGGAATCCAAAACATCCAAAACCTGATCAAATTGATCCTTTTTACCCTAaaTTActcgagTTAGTC | - This study |
| - 5 | - GDH2-VerF | - CACATAGAGTATGCATGCAC | - This study |
| - 6 | - GDH2-VerR | - GATTCAGCATCAACAGTGTC | - This study |
| - 7 | - GDH2-GFPTop | - GTTTACTCTAGAGGAATCGATTCTAATCCTGCTAAATTTTTGGAATTTATCAGTTCTATTAGAAAGGATTTTATTCAAAAGGGATTGCTCAAGTATGGTGCTGGCGCAGGTGCTTC | - This study |
| - 8 | - GDH2-GFPBot | - AAGCAAACTTTAAATAAATAATTATAAATAGAATTTTTGAAAATCAAGCATTTTCTCATAATTATAGATAAATCTCTAAACGTATTTGAAACAACCTCTGATATCATCGATGAATTCGAG | - This study |
| - 9 | - GDH2-GFPverrev | - CTTCGGGCATGGCACTCTTG | - This study |
| - 10 | - p91_FS95 | ggcatagctgaaacttcggc | [15] |
| - 11 | - p112_5'ADH1test | ACAATATTTGATAGAGAC | [15] |
| - 12 | - p113_3'ADH1test | TTGAATCTACGAGACTC | [15] |
| - 13 | - FS344 | CTAGAGTTTACTCGATCGACAC | This study |
