# Supplementary material for: Glutamate dehydrogenase (Gdh2)-dependent alkalization is dispensable for escape from macrophages and virulence of Candida albicans
Source: PLoS Pathog. 2020 Sep 16;16(9):e1008328. doi: 10.1371/journal.ppat.1008328 (PMC7521896; doi:10.1371/journal.ppat.1008328)
Supplement: Attachment — Submitted filename: Resub_Letter.pdf [file ppat.1008328.s023.pdf]

Stockholm University
Department of Molecular Biosciences,
The Wenner-Gren Institute

Stockholm 2020-02-28

PLoS Pathogens

**Scott Filler**	**Kasturi Haldar**	**Michael Malim**
Section Editor	Editor-in-Chief	Editor-in-Chief

Re: PPATHOGENS-D-20-00074

Dear Scott, Kasturi and Michael,

Thank you for reconsidering the initial negative decision on our manuscript "Glutamate dehydrogenase (Gdh2)-dependent alkalization is dispensable for escape from macrophages and virulence of *Candida albicans*" (PPATHOGENS-D-20-00074). My co-authors and I are pleased that *PLoS Pathogens* will send this manuscript out for peer review. As suggested, we have taken the opportunity to adjust the discussion section to enhance clarity, and hopefully make the significance of our work clearer.

We understand that although our manuscript will now be sent for review, ultimate acceptance for publication is dependent on our determination of whether phagosomal alkalization is affected by inactivation of Gdh2. As I clearly described in my letter of appeal, based on our finding that Gdh2 is dispensable for hyphal growth and the recent work indicating that hyphal growth is the cause of phagosome alkalization (Westman et al. 2018), we do not anticipate to see an affect. Consequently, seeing an affect cannot remain or be a criteria for determining acceptance.

With this said, we are acquiring the necessary reagents and setting up the microscopy methods required to measure the phagosomal pH. We anticipate to have results in a time-frame compatible with the review process. We will take the demand for measuring phagosomal alkalization as a critical test of Westman et al. 2018, and not our own findings. The only notable result would be if we fail to observe alkalization, which would then question the model of Westman et al, 2018, which is fair enough.

Again, I am happy that colleagues in the field will be given the opportunity to review our work and to critically evaluate currently held dogma.

Respectfully,

Per O. Ljungdahl, Ph.D.
Professor of Cell Biology

Postal address:
Stockholm University
106 91 Stockholm, Sweden

Visiting address:
Arrheniuslaboratorierna F466
Svante Arrhenius väg 20B

Phone: +46 8 16 41 01
Fax: +46 8 16 42 09
E-mail: per.ljungdahl@su.se
