# Supplementary material for: Glutamate dehydrogenase (Gdh2)-dependent alkalization is dispensable for escape from macrophages and virulence of Candida albicans
Source: PLoS Pathog. 2020 Sep 16;16(9):e1008328. doi: 10.1371/journal.ppat.1008328 (PMC7521896; doi:10.1371/journal.ppat.1008328)
Supplement: Attachment — Submitted filename: Point_to_Point_Gdh2.pdf [file ppat.1008328.s024.pdf]

Point-to-Point Replies to Reviewer Comments

Reviewer #1:

Part I – Summary This manuscript describes a role for the C. albicans Glutamate dehydrogenase (Gdh2) in alkalization of the external environment, and surprisingly shows that this function is dispensable for escape from macrophages and virulence. This was unexpected because a previous model by Lorenz et al proposed that growth on amino acids in the phagosome would lead to ammonia release and alkalization, which would stimulate hyphal growth and escape from the phagosome. Therefore, it was surprising that that deletion of GDH2, which prevented alkalization in vitro, did not affect escape from macrophages or virulence. One strength of the manuscript is that the data provide strong support for a role for Gdh2 in alkalization for cells grown on amino acids in vitro. This work also synergizes with previous work by this group of investigators showing that proline catabolism, which goes through Gdh2 and glutamate, is important for growth on amino acids and alkalization. Another interesting conclusion was the demonstration that glucose could repress mitochondrial function, and GDH2 expression, preventing alkalization of the environment when amino acids were present. However, a weakness is that the pH of the phagosome was not measured. It is not clear if metabolism of other metabolites by gdh2 mutant cells still leads to alkalization of the phagosome. For example, GlcNAc, alpha-ketoglutarate, and pyruvate have also been observed to contribute to alkalization.
We have now followed phagocytosis of *C. albicans* by macrophages in real time using live cell imaging and measured phagosomal pH using pHrodo (data presented in Fig. 6). Consistent with the report by Westman et al. (2018, mBio), the phagosomes containing the WT or *gdh2*-/- cells are rapidly acidified and hyphal formation by phagocytized *C. albicans* initiates at acidic pH. Consequently, the possibility that alkalization may result from the metabolism of other metabolites, e.g., GlcNAc, alpha-ketoglutarate, or pyruvate, is unlikely. Furthermore, the *gdh2*-/- mutant exhibited a more pronounced alkalization defect as compared to the *stp2*Δ/Δ mutant (Fig. S2), previously reported to be defective in phagosomal escape. Even at a high starting OD of 5.0, *gdh2*-/- mutants fail to alkalinize the medium after 24 h (Fig. S2). However and important to note, contrary to Westman et al, the phagosomal compartment remains acidic even as fungal cells sustain hyphal growth leading to the eventual lysis of the macrophage.

Part II – Major Issues: Key Experiments Required for Acceptance

1. The gdh2 mutant strain was not complemented by reintroduction of the wild type GDH2 gene. This control is especially important, considering that the authors come to a different conclusion than previous studies.
We have now reintroduced *GDH2* into its endogenous locus creating *GDH2/gdh2-* heterozygous strains in both SC5314 and *cph1*Δ/Δ *efg1* Δ/Δ backgrounds (**Fig. S2**). The *gdh2-* allele is fully recessive and heterozygous strains exhibit wildtype phenotypes.

2. As mentioned above, the pH of the phagosome was not measured, so it is unclear if there are alternative pathways that can lead to alkalization in the absence of GDH2.
We have monitored phagosomal pH using the pH-sensitive pHrodo dye. No differences in pH were observed between phagosomes containing WT or gdh2-/- cells. In both instances, phagosomes became progressively acidified following phagocytosis. Increasing the MOI of the wildtype and *gdh2*-/- to obtain macrophages containing multiple phagocytized cells had no effect and did not reduce the ability of macrophages to acidify and maintain low phagosomal pH. This is consistent with the high proton pumping capacity of the V-ATPase.

3. Gdh2-GFP looks different in Figure 3A, than 2A. It looks cytoplasmic in Fig. 3A rather than mitochondrial. This raises questions about whether Fig. 3A is accurately reflecting the levels and localization of Gdh2.
The micrographs shown in Fig. 2A/3A were obtained using a confocal microscope. Fig. 2A includes images of cells grown in liquid culture whereas Fig. 3A includes cells grown on agar slabs. We have noted that when the fluorescent signals are high, the patterns exhibit a more diffuse pattern, when in fact it is exclusively localized in the mitochondria; note how, in Fig. 3A, the signal gains intensity from 2-4 h exhibiting a classical mitochondrial distribution.

Part III – Minor Issues: Editorial and Data Presentation Modifications

1. The text should be modified to indicate that the lack of an essential role for in macrophage escape is not the same as saying it can't contribute to escape along with other pathways.
Our data clearly show that Gdh2 is not essential for macrophage escape. There may be synthetic defects if combined with other mutations, however, since we observe no differences between WT and *gdh2*-/- strains in macrophage escape, and hyphal growth clearly is initiated and sustained at low phagosomal pH, we have no basis to think that Gdh2 contributes to other pathways.

2. In Figure 6, was the CFU assay done with macrophages infected with a single strain or mixed culture? If a WT and a gdh2-/- cell are in the same macrophage, the WT cell could alkalinize the phagosome and protect the gdh2-/- mutant.
We clarified this in the text, and have repeated the experiments using time-lapse microscopy to separately follow the phagocytosis of each individual strain (new Figure 5). The competition experiment highlights all conditions since we captured macrophages with either of the strains and macrophages with both strains. Supplementary videos (SV3 and SV4) to show behavior of each strain when co-cultured with macrophage are now included.

3. It is not obvious that Gdh2-GFP is getting brighter in Figures SV1 and SV2 and Figure 5. The level of Gdh2-GFP should be quantified to support the conclusion Gdh2 is induced.
We do not agree with the reviewer. Consistent with immunoblot analysis of protein levels in extracts prepared from cells shifted from high to low glucose, the GFP signal becomes obviously brighter in phagosome, any transient decreases in signal strength seen is due to shifts in focal plane as the macrophages move and change their shape.

4. The authors should comment on why there was only modest effect on growth of gdh2-/- mutant on proline in Fig. S1C? If Gdh2 is only partially necessary for growth on proline, does this indicate a gdh2-/- mutant could still contribute to alkalization in a phagosome, albeit weaklier?
The data indicate that the steps of proline metabolism leading to glutamate yield sufficient energy for growth, independent of subsequent metabolism of glutamate as a nitrogen source. Consequently, similar growth is expected in SPD and SED, which we indeed observe. Due to the fact that *C. albicans* has a mitochondrial NADH dehydrogenase coupled to proton translocation means the 5 ATP molecules can be generated by the metabolism of proline to glutamate.

5. Does it matter which medium the C. albicans cells were grown in before they were used to infect macrophages? Residual carbon, nitrogen, and energy sources may dominate in the first stages of growth in the macrophage. This could make it more difficult to assess the role of Gdh2.
This is a good point; we have actually examined cells from early log phase and mid/late log phase YPD cultures. We observe hyphal formation in HBSS within a 4 h period when mid/late log phase cells are used, early log phase cells exhibit significant delayed hyphal growth.

6. How was FITC staining done? Please provide additional information. E.g. was it crosslinked onto the cells? Taken up by endocytosis? The reason I am asking is that Figure 4B looks weird. The entire cell is stained. I was expecting new growth should not be stained if the FITC is cross-linked.
As we noted in Part II, point 3, when the fluorescent signal is high, it can give the impression of a diffuse pattern, a phenomenon particularly evident in confocal images. Consequently, the FITC appears associated with hyphae, especially in the early time periods. In revising the manuscript with the inclusion of additional data, we have chosen to remove the images in question.

7. Lines 172-174. This sentence should be clarified that Gdh2 is primarily, but not solely, responsible for ammonia production when cells were grown on glutamate.
In our hands, *gdh2*-/- cannot alkalinize medium containing amino acids (not just glutamate) even when starting with high cell densities and cultures are grown for 24 h. In fact, we used the no growth and alkalization phenotype to identify reconstituted strains, which under the selection conditions are strictly

coupled. As we mentioned earlier, the *gdh2*-/- mutant even exceeded the alkalization defect of *stp2*Δ/Δ mutant, previously reported to have a strong alkalization defect (Fig. S2).

Reviewer #2:

Part I – Summary This manuscript describes an investigation of the factors required by C. albicans for alkalization of its environment. Alkalization of the phagosome environment upon engulfment of C. albicans has previously been hypothesized to induce the yeast to hyphal switch facilitating C. albicans escape from immune cells and therefore of high importance to pathogenicity. Here, in agreement with a previous study (Westman et al, 2018), the authors show that alkalization is not required for hyphal induction inside the phagosome. The authors demonstrate that a mutant strain lacking Gdh2 glutamate dehydrogenase is defective in alkalization of a synthetic amino acid containing medium in contrast to wild type cells. GDh2-GFP was localized to mitochondria and expression was repressed in high concentrations of glucose but induced upon macrophage engulfment. The alkalization defect did not result in a morphogenesis defect in vitro and in macrophage co-culture assays. The ghd2 mutant was also virulent in a mouse and Drosophila infection models. In S. cerevisiae Ghd2 is also glucose – repressed and mitochondrial.

Part II – Major Issues: Key Experiments Required for Acceptance

A key experiment as set out in the authors' covering letter is to show that the ghd2 mutant is incapable of alkalizing the macrophage phagosome. This will strengthen the evidence presented and clearly confirm the role of Ghd2 in this process in vivo ; reinforcing the in vitro measurements of pH changes presented in the manuscript.

We have used the pH-sensitive dye (pHrodo) to monitor the pH of the phagosomal compartment when *C. albicans* cells are engulfed by macrophages. Our data clearly show that phagosome acidification occurs rapidly with either WT or gdh2-/- cells. Similar to Westman et al 2018, we observed that hyphal growth initiates and continues within the phagosome, which remains acidic until macrophage lysis. We have added several time-lapse movies that demonstrate this phenomenon.

Part III – Minor Issues: Editorial and Data Presentation Modifications

Supplementary video 3 very rapidly goes out of focus so is not informative.

The previously submitted supplemental video 3a (SV3a) contained time-lapse images, starting in focus, but focus drifted, which was observed at 1 hr, at which point the microscope was refocused, and remained in focus for the remainder of the experiment (SV3b).These videos document the co-culturing wildtype and gdh2-/- together with macrophages. We feel that it is important to include both videos so that readers can see the t=0 time point. The issue with focus is not relevant to interpretation of the extended time-lapse (the videos now are SV5a and SV5b). Also, we have repeated the time lapse experiments to individually examine co-cultures of WT and gdh2-/- strains with BMDM (see new videos V3 and V4 that were used to prepare new Fig. 5A and 5B).

Line 192 strain

Text adjusted

Line 200. Colonies were grown on solid……and the levels of volatile ammonia produced were measured.

Text adjusted

Line 218 medium

Text adjusted

Line 269 define NCR

We have adjusted the text to define NCR.

Line 310 macrophages Phagocytized and phagocytized (line 325) both used in text, keep consistent

We have adjusted the text to consistently use *phagocytized*.

Reviewer #3
Part I – Summary This manuscript characterized the role Candida albicans Gdh2 in alkalization under conditions of amino acids as a sole carbon source. The finding of a requirement for production of NH_3 under these conditions in interesting, particularly as disruption has no impact on phagocytosis or virulence.

Strengths:
Inclusion of the GFP-strain to track Gdh2 to mitochondria
The inclusion of two animal models and multiple experiments to measure virulence
Most experiment use multiple lines of evidence

Limitations:
It is unclear gdh2 is responsible for NH_3 production in the phagosome
It is unclear if disruption of gdh2 influences the phagosome pH.

Part II – Major Issues: Key Experiments Required for Acceptance
Figure: 1C and 1D: For figure 1D, it appears that YNB +CAA +0.2% glucose was used from the text and should be included in the legend. It is not clear how well the gdh2 mutant grow in this media. If it is not growing well, this may impact NH_3 production. A growth control should be used.
The figure legend now indicates the media used in Fig. 1D, which was YNB +CAA +0.2% glucose. It is true that alkalization is cell density dependent and that *gdh2*-/- has severe growth defect on amino acid medium in the absence of additional carbon source. We attribute the need of an additional carbon source to supply sufficient 2-carbon acetyl-CoA to enable TCA cycle, which is important for glutamate synthesis from alpha-ketoglutarate. When we add non-repressing levels of an additional carbon source (0.2% glucose or 1% glycerol), gdh2-/- cells grow, albeit more slowly than WT, and even at a very high cell densities, *gdh2*-/- cultures remain acidic (Fig. S2, Fig. S5). Consequently, Gdh2 is the primary source of alkalizing NH_3 when cells grow in media with CAA.

Line 302: Can the authors add data showing that the phagosome is or is not alkalinized when the gdh2 mutant is engulfed?
Yes, we have now monitored the pH and the results are now included.

Part III – Minor Issues: Editorial and Data Presentation Modifications
Figure S1: Why was the screen performed? Why were the mutants with the correct insertion not initially identified by PCR?
The pre-screening approach is rational and is based on the finding that arginine cannot be efficiently utilized by proline (*put*) mutants, thus, it is likely that the lack of Gdh2 (which is downstream of Put) would also show a similar phenotype. CRISPR mutagenesis problem is sometimes difficult to troubleshoot (sgRNA design, RT design, etc.) so screening by phenotype would allow us to identify strains carrying the desired inactivating mutations. Our approach was to first check growth phenotypes under selective conditions and subsequently verify putative clones from the original (non-selective) plate (including controls) by PCR and restriction analysis. Nourseothricin resistant transformants that are negative for both the mutant phenotype (alkalization positive) and PCR (*GDH2*+/+) were saved as controls (See strain list CFG283 and CFG347).

The authors should expand on how the growth media and conditions in the current study do or do not represent phagosome conditions.
Since we are studying metabolic pathways conferring alkalization, it is imperative to understand the effect of each component on cellular physiology. The initial recipe that we used in our alkalization is YNB+CAA+BCP (pH = 4) without ammonium sulfate. We omitted ammonium sulfate that was used by Vylkova et al. since the high level of ammonium (38 mM) can potentially repress NCR sensitive genes, which inadvertently could affect our observations. Also, this level of ammonium is not present *in vivo* within phagosomes. Our data showing the sensitivity of Gdh2 expression to glucose and our finding that Gdh2 levels are induced upon phagocytosis, is consistent with the notion that glucose is low in phagosomes.

In our co-culture phagocytic assays, we use HBSS as media since macrophages remain healthy for several hours and hyphal growth of non-phagocytized cells is delayed compared to that observed in standard cell culture medium (DMEM or RPMI). The use of HBSS medium mimics the osmolarity in interstitial fluid that baths the macrophages in situ in hosts.

Figure 2A: Include a glucose-rich condition.
We have included high-glucose in our revised figures – see immunoblot (**Fig.3C**) and fluorescence microscopy (**Fig. S6**). Glucose represses the Gdh2 expression.

Figure 2B: What is the impact of antimycin alone on the media? I assume that these experiments use the same pH indicator as 1B, but this should be added to the legend.
Antimycin A and the ethanol carrier do not affect pH. Yes, we used BCP, the legend has been adjusted as recommended.

Lines 226-234: The description of the figure does not appear to match Figure 2. I don't see right and left panels or the gdh2 mutant in Figure 2B.
Thanks! Yes, (-) indicates the sterile medium.

Figure S2A: For the right panel, clarify the difference between Cm – and Cm 0. I assume that no organism was added to the Cm-, but this should be clarified.
Yes, (-) indicates the sterile medium, the text has been adjusted.

Line 245: The authors should describe why different results were found for antimycin and chloramphenicol
Antimycin A is more potent than chloramphenicol and acts directly on existing Complex III. Chloramphenicol acts on mitochondrial translation so the effect depends on gradual reduction of existing proteins, and consequently, is not as rapid as direct inhibition of Complex III by Antimycin A. This should explain why acute inhibition of alkalization in highly dense culture does not work. We have adjusted the text.

Line 270: Clarify NCR
This is now clarified in the text.

Figure 5A and B and supplemental video: Is there a peak fluorescence that is reached prior to the end of the experiment? If so, can the authors speculate as to why this is? Are the phagocytes dying and the glucose repression ending?
We added a supplementary figure (S6) to address this. Gdh2-GFP increases rapidly when there is no glucose. The fact that the GFP level increased following engulfment by macrophage suggest that glucose is limiting in phagosomes, which is consistent with available information. The apparent reduction in GFP could be due to increase in cell volume when hyphae become longer or when there is a sudden shift in focal plane during continuous imaging.

Line 337: Clarify the statement, as alkalization was not measured in the phagosome.
We have now monitored the phagosomal pH. This is now an integral part of the paper.

Line 378: The authors should avoid discussing the unpublished data, or include the data.
The relevant data is now included in the supplementary figure (Fig. S2).

Line 448: Clarify if these conditions for filamentation were the same as the ones used in the current investigation and consider having a supplemental figure with the strains.
We have used Lee's and Spider media, standard media that are known to facilitate robust hyphal growth. The point here is that *gdh2-/-* mutants exhibit similar filamentous growth as WT on these media.
