# Supplementary material for: Glutamate dehydrogenase (Gdh2)-dependent alkalization is dispensable for escape from macrophages and virulence of Candida albicans
Source: PLoS Pathog. 2020 Sep 16;16(9):e1008328. doi: 10.1371/journal.ppat.1008328 (PMC7521896; doi:10.1371/journal.ppat.1008328)
Supplement: Attachment — Submitted filename: Points_Reviewers comments_200808.pdf [file ppat.1008328.s025.pdf]

Point-to-Point Replies to Reviewer Comments

We thank the reviewers for their thorough critical review and for providing constructive comments. Their input has helped to improve our manuscript.

Part I – Summary
Please use this section to discuss strengths/weaknesses of study, novelty/significance, general execution and scholarship.

Reviewer #1: A major improvement was the inclusion of new data demonstrating the acidification of the lysosomes following C. albicans phagocytosis. The authors used a new approach of crosslinking a pH sensitive dye to the cells (pHrodo). A strength of this approach is that the fluorescence increases as the ambient pH gets lower. This avoids concerns that factors such as photobleaching could interfere.

However, there are some minor issues with the manuscript that need to be addressed. In regard to the comments below concerning Gdh2-GFP, the authors should be clearer about what are the limits of their conclusions regarding the levels of Gdh2-GFP. Also, they should explain why in some experiments Gdh2-GFP does not appear to localize to the mitochondria.

To unambiguously locate the subcellular origin of Gdh2-GFP signal, we repeated the colocalization experiment with MitoTracker (MTR) using confocal microscopy (Airyscan). This confirmed that Gdh2 co-localized to mitochondria (see revised **Fig. S6**). We note that the apparent deviation from the canonical mitochondrial pattern in the figures provided for review could be due to several technical factors: 1) the time-lapse acquisition uses reduced exposure time to retain cell viability and to avoid bleaching, 2) as signal intensities significantly increase they often appear more diffuse, and 3) the resolution of the images uploaded for review had a reduced quality (full resolution images accompany this final resubmission). As for the derepression of Gdh2 in engulfed fungal cells, we added new data showing that Gdh2-GFP expression is indeed higher in engulfed fungal cells compared to external cells. We accomplished this by using a reporter strain that expresses both Gdh2-GFP and free RFP, which is under the control of the strong constitutive *ADH1* promoter. We used RFP as a control expression to allow us generate a more quantitative comparison between engulfed and external fungal cells.

Reviewer #3: The authors have performed the additional requested studies and addressed the comments of the reviewers.

We thank the reviewer for previous suggestions that have improved our manuscript.

Part II – Major Issues: Key Experiments Required for Acceptance
Please use this section to detail the key new experiments or modifications of existing experiments that should be absolutely required to validate study conclusions.

Reviewer #1: (No Response)

Reviewer #3: (No Response)

Part III – Minor Issues: Editorial and Data Presentation Modifications
Please use this section for editorial suggestions as well as relatively minor modifications of existing data that would enhance clarity.

Reviewer #1:
1. Lines 380-381,393, 514 and 1048. The manuscript refers to a supplemental video SV12, but SV12 was not included.

We have included the video as SV12, apologies for the omission.

2. The authors complemented the gdh2-/- strain by reintroducing a DNA fragment carrying the wild type GDH2 gene. Was it marked in any way to distinguish it from a pre-existing gdh2-/GDH2 strain?

We used CRISPR/Cas9 to generate the homozygous *gdh2*-/- mutants used in our studies. Diagnostic for the successful inactivation of *GDH2* is the gain of an *Xho*I restriction site into both alleles via the repair template (RT). Strains CFG279 and CFG354 were confirmed homozygous *gdh2*-/- mutants (both alleles have the *Xho*I site). These mutant strains were transformed using a PCR amplified wildtype *GDH2* gene fragment with homologous sequences flanking the *Xho*I insertion site; transformants were selected by growth on YNB+CAA, a condition that requires Gdh2 activity. Subsequent analysis revealed that a single *Xho*I site in one of the *gdh2* alleles was lost, confirming the desired integration into the

correct locus and restoration of *GDH2*. We would like to point out that prior to the genetic reconstitution, we did not have access to a *GDH2/gdh2*$^{-}$ heterozygous strain. As indicated in the text, the growth and alkalization phenotypes of *gdh2*-/- in YNB+CAA were used as a selection marker. The presence of the *Xho*I-negative allele correlated fully with the Alk^{+} phenotype (Fig. S3), further indicating the recessive nature of *gdh2*$^{-}$*.* The absence of growth of cells transformed with a negative control fragment from pJA21 (P_{ADH1}-*RFP-caSAT1*), which normally exhibits a high transformation efficiency, is consistent with growth specifically due to restoration of *GDH2*.

3. Fig. 3A. The Gdh2-GFP signal goes up, but it does not look mitochondrial

We updated Fig. 3A with a high-resolution image including an inset to show that the signals are localized to mitochondria.

4. Fig S6. The Gdh2-GFP signal goes up, but it does not look mitochondrial

We updated Fig. S6 with a high-resolution image to show a clearly mitochondrial pattern of Gdh2-GFP expression. We also included control data to show that the Gdh2-GFP signal in one of the high-expression conditions (i.e., no addition, T = 4 h) co-localizes with MitoTracker.

5. Fig. 4B and 4C. Not clear that the Gdh2-GFP signal is increasing. How was this quantified.

The inclusion of these time-lapse movies is to provide readers with a qualitative evidence that Gdh2-GFP expression is induced in fungal cells upon their phagocytosis by macrophages. We have now quantified the increase in expression using a *GDH2-GFP*/P_{ADH1}-*RFP* reporter strain. This approach enabled us to gain an appreciation for the increased GFP signal in macrophage-internalized relative to external fungal cells. The details and new data are described in the revised text and Fig. 4C (new panel).

6. SV2 Not clear that the Gdh2-GFP is increasing until the hyphae break out of the macrophage. How was this quantified?

Same explanation as in 5. There is a noticeable increase in GFP signal around 20-40 min, however, due to the high green autofluorescence in BMDM the signal appears weak. To clarify our findings presented in Fig. 4, we present the time-lapse merely as a supplementary video (SV2).
