# Supplementary figures and images for: Correction: Glutamate dehydrogenase (Gdh2)-dependent alkalization is dispensable for escape from macrophages and virulence of Candida albicans
Source: PLoS Pathog. 2021 Aug 30;17(8):e1009877. doi: 10.1371/journal.ppat.1009877 (PMC8405231; doi:10.1371/journal.ppat.1009877)

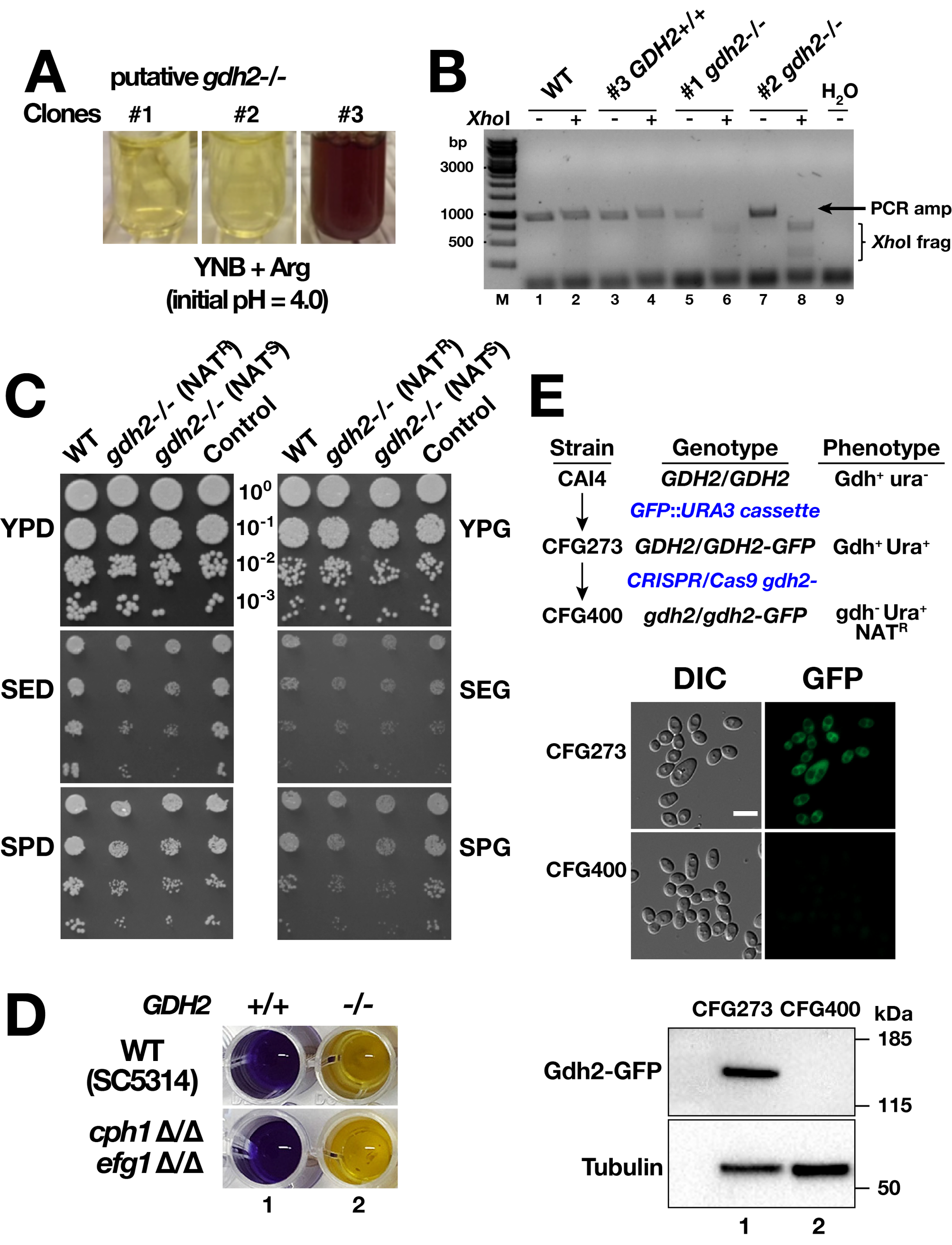

Supplement: S1 Fig — (A) A purified KpnI/SacI fragment from pFS108, harboring GDH2-specific sgRNA, and PCR generated repair template (RT) were introduced into wildtype strain SC5314 by electroporation. NouR transformants were pre-screened in YNB+Arg medium containing the pH indicator bromocresol purple; the initial pH was 4.0. Three NouR colonies were picked for further analysis. Clones #1 and #2 grew poorly and were unable to alkalinize the media; clone #3 grew and alkalinized the media. (B) Genomic DNA, isolated from the three clones, was used as template for PCR amplification of the targeted GDH2 locus; ddH2O was used as negative control. Restriction of the amplified ≈900 bp fragment by XhoI is diagnostic for successful mutagenesis (primers p5/p6; S2 Table). Strains, clone #1 (CFG277) and clone #2 (CFG278) carry inactivated gdh2-/- alleles. (C) GDH2 is not essential but required for robust growth on glutamate or proline as sole nitrogen source. Five microliters of serially diluted wildtype (SC5314), gdh2-/- NATR (CFG277), gdh2-/- NATS (CFG279), and control (CFG182) cells were spotted on yeast peptone (YP), synthetic glutamate (SE) and synthetic proline (SP) media containing either 2% glucose (YPD, SED, SPD) or 1% glycerol (YPG, SEG, SPG) as carbon source. The plates were incubated for 48 h at 30°C and photographed. (D) Fresh colonies of SC5314 (PLC005; WT), CFG279 (gdh2-/-), CASJ041 (cph1-/- efg1-/-) and CFG352 (cph1-/- efg1-/- gdh2-/-) were individually resuspended in YNB+CAA medium and incubated for 24 h at 37°C. (E) The insertion of GFP in strain CFG273 (GDH2-GFP) was verified by PCR, the expected 1695 bp fragment was amplified using primers (p24/p25; S2 Table); strain CAI4 served as untagged control (middle left panel). CFG273 was transformed with the CRISPR/Cas9 cassette to inactivate GDH2. Putative gdh2-/- clones were identified as described and verified by PCR-RD (p13/p6; S2 Table) resulting in strain CFG400. Strains CFG273 (GDH2/GDH2-GFP) and CFG400 (gdh2/gdh2-GFP) were grown [file ppat.1009877.s001.tif]

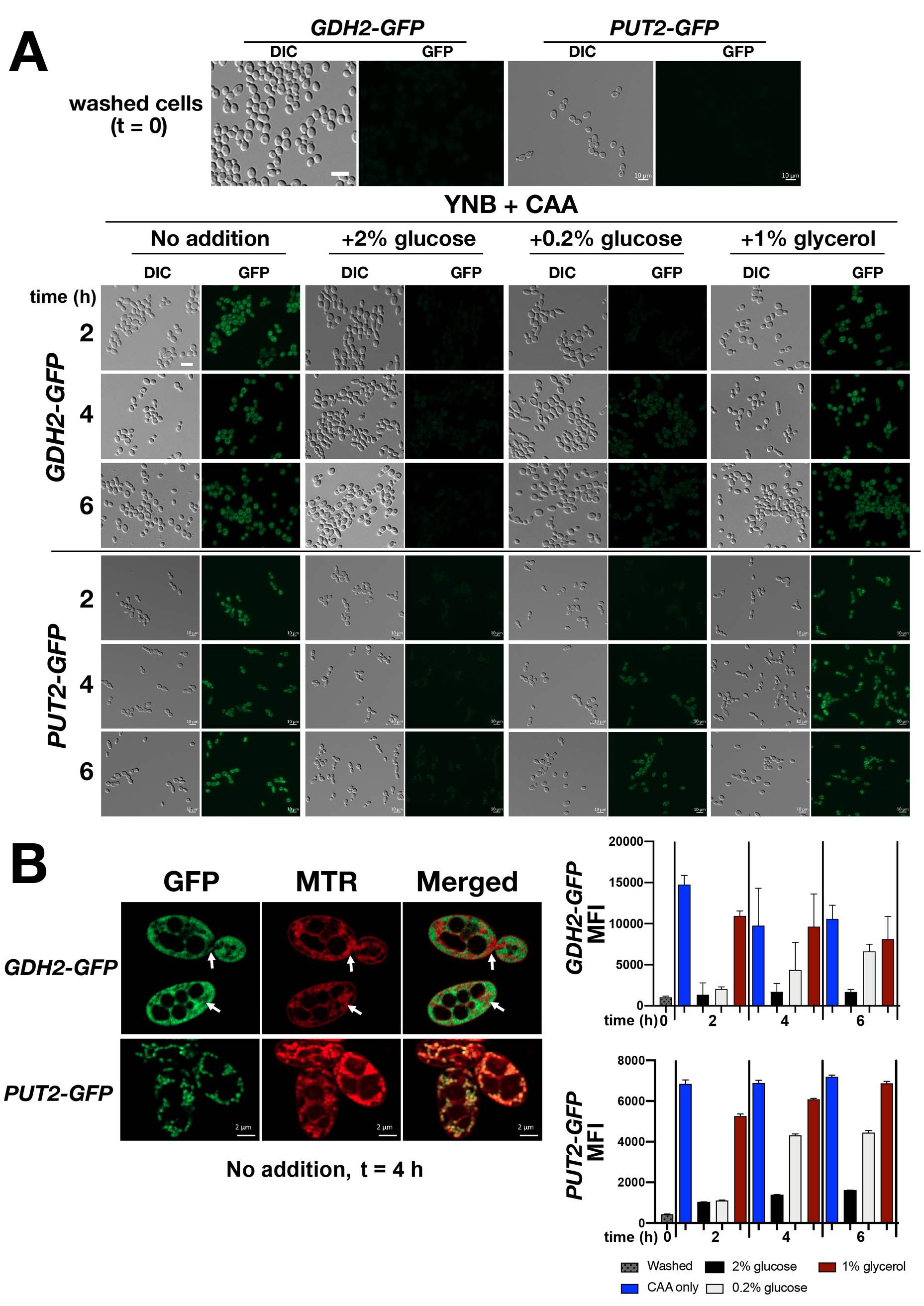

Supplement: S6 Fig — (A) Cells expressing GDH2-GFP (CFG273) were collected from YPD overnight cultures, washed, and then diluted in liquid YNB+CAA with or without the indicated concentrations of glucose or glycerol at OD600 ≈ 2.0 and then incubated under aeration at 37°C. Cells were harvested at the indicated time points and then immediately washed with ddH2O for microscopic examination of Gdh2-GFP expression. Representative images of cells from each condition with their relative expression of Gdh2-GFP are shown. Results obtained for the strain CFG219 (PUT2-GFP) are shown for comparison. Scale bar = 10 μm. (B) (Left) Cells collected from no addition (T = 4h) were stained with MitoTracker Deep Red (MTR; 200 nM) and then observed by confocal microscopy (LSM800) using the Airyscan detector. The Gdh2-GFP and MTR signals are mutually exclusive whereas the Put2-GFP colocalizes with MTR; Scale bar = 2 μm. (Right) Quantification of mean fluorescence intensity (MFI) from at least 3 biological replicates per condition (≥150 cells/replicate) are shown. (TIF) [file ppat.1009877.s002.tif]
